# Cytokine production in an *ex vivo* model of SARS-CoV-2 lung infection

**DOI:** 10.3389/fimmu.2024.1448515

**Published:** 2024-10-21

**Authors:** Daria A. Vorobyeva, Daria M. Potashnikova, Elena V. Maryukhnich, George I. Rusakovich, Anna V. Tvorogova, Anna I. Kalinskaya, Natalia V. Pinegina, Anna V. Kovyrshina, Inna V. Dolzhikova, Alexander B. Postnikov, Fedor N. Rozov, Tatiana N. Sotnikova, Dmitry Yu. Kanner, Denis Yu. Logunov, Alexander L. Gintsburg, Elena J. Vasilieva, Leonid B. Margolis

**Affiliations:** ^1^ Laboratory of Atherothrombosis, Cardiology Department, Federal State Budgetary Educational Institution of Higher Education (FSBEI HE) “Russian University of Medicine” of the Ministry of Health of the Russian Federation, Moscow, Russia; ^2^ I.V. Davydovsky Moscow City Clinical Hospital, Moscow Department of Healthcare, Moscow, Russia; ^3^ Federal Government Budgetary Institution “The National Research Center for Epidemiology and Microbiology Named After Honorary Academician N.F. Gamaleya” of the Ministry of Health of the Russian Federation, Moscow, Russia; ^4^ HyTest Ltd, Turku, Finland; ^5^ Moscow City Oncology Hospital No 62, Moscow, Russia; ^6^ Faculty of Natural Sciences and Medicine, Ilia State University, Tbilisi, Georgia

**Keywords:** *ex vivo* model, lung explant *ex vivo* culture, SARS-CoV-2, COVID-19, cytokines, immune response, antiviral response

## Abstract

**Introduction:**

The mechanisms of the SARS-CoV-2-triggered complex alterations in immune cell activation and production of cytokines in lung tissue remain poorly understood, in part because of the limited use of adequate tissue models that simulate the structure and cell composition of the lung *in vivo*. We developed a novel *ex vivo* model of SARS-CoV-2 infection of lung explants, that maintains the intact tissue composition and the viral load for up to 7–10 days. Using this model, we studied cytokine production during SARS-CoV-2 infection.

**Materials and methods:**

Lung tissue was monitored for viability and cell composition using flow cytometry and histological analysis. SARS-CoV-2 infection was verified immunohistochemically, viral loads in tissue and culture medium were monitored by qPCR. A panel of 41 cytokines was measured in culture medium using xMAP technology.

**Results:**

The explant lung tissue was viable and maintained viral infection that influenced the cytokine production. Elevated concentrations of G-CSF, GM-CSF, GRO-a, IFN-g, IL-6, IL-8, IP-10, MCP-3, MIP-1a, PDGF-AA, and VEGF, and decreased IL-1RA concentration were observed in infected tissue compared to non-infected tissue.

**Discussion:**

Our results generally reflect the data obtained in COVID-19 patients. GRO-a, IFN-g, IL-6, IL-8, MCP-1, MCP-3, and RANTES correlated with the viral load, forming a distinct pro-inflammatory cluster. Thus, our lung *ex vivo* model faithfully reproduces some aspects of cytokine alterations in COVID-19 patients at an early disease stage, making the investigation of SARS-CoV-2 infection mechanisms more accessible and providing a potential platform for antiviral drug testing.

## 1 Introduction

The new coronavirus disease COVID-19 has demonstrated a number of physiologic and metabolic abnormalities in the human organism that are associated with viral infection at both the systemic and the organ level. The systemic alterations, detected from blood plasma analysis, include a change in key metabolites and cytokine levels of the patients that might result in a cytokine storm syndrome – a severe systemic response to inflammation (1–4).

At the organ level, SARS-CoV-2 infection affects lung cells (especially type II alveolocytes), which show a high susceptibility to the SARS-CoV-2 both *in vivo* and *in vitro*, and probably constitute the primary trigger of inflammation (5–7). COVID-19 is often associated with pneumonia – a common inflammatory lung disease that has acquired a priority position in medical research as one of the main causes of death in severe COVID-19 cases (8). Pneumonia is characterized by damage to lung tissues that can occur both because of the direct virus action and of the excessive and uncontrolled immune response (9).

An immune response to SARS-CoV-2 *in vivo* includes a cooperative action of a wide range of immune cells, such as tissue-resident and infiltrating macrophages and dendritic cells that are responsible for phagocytosis and production of pro-inflammatory cytokines (10, 11), neutrophils that can produce NETs (neutrophil extracellular traps), trigger coagulation and thrombosis and participate in cytokine storm (12), and T cells that may boost the local inflammatory microenvironment and ensure persistent anti-viral protection *in situ* by IFNγ (13, 14). All of these cell types can facilitate virus eradication, but can lead to immune pathologies due to dysregulated immune responses (15). The effects of these cells are mediated by various stimuli, primarily cytokines, that have a prominent role in orchestrating cell populations in the inflammatory response to SARS-CoV-2 infection (16). Their action can both promote the resolution of disease and dysregulate the inflammation and stimulate life-threatening conditions (17, 18).

The mechanisms of the SARS-CoV-2-triggered complex alterations in immune cell activation, inactivation, production of cytokines, etc., have been poorly understood so far, in part because of the lack of adequate models that simulate the structure and cell composition of lung tissue *in vivo* and thus fail to faithfully reflect the complexity of the *in vivo* pathology. For instance, cell culture models provided important information regarding viral infection, but did not account for the 3D tissue organization and diverse cell content (19, 20). Organoid models were more physiological and diverse, yet they did not properly represent the lung immune microenvironment (21, 22). Animal models also did not faithfully represent some important aspects of human immune system and may not always allow to separate the local and the systemic immune responses (23). Hence, the lung explant models have several advantages, such as preserved organ structure, diversity of cell types, including the immune cells and a good potential to provide a relatively simple, cost-effective and accurate way to mimic the immune responses *in situ* observed during respiratory infections, such as COVID-19-associated pneumonia (24, 25). Here, we further developed a clinically relevant model of human lung explants that allows to mimic the initial events of SARS-CoV-2 infection and assess the viral effects on cytokine production.

## 2 Materials and methods

### 2.1 Ethics committee

The study was approved by the Moscow City Ethics Committee (protocol № 50/69_13.10.2020) of the Research Institute of the Organization of Health and Healthcare Management and performed according to the Declaration of Helsinki.

### 2.2 Specimens and reagents

We analyzed 18 post-mortem reference lung specimens obtained from individuals who died from COVID-19-associated pneumonia in April and May 2020. The clinical data on the patients are presented in **Supplementary Table S1**. The lung tissue specimens were stored in RNAlater RNA stabilization reagent (Qiagen, US) at -20°C for SARS-CoV-2 viral load assessment and as FFPE blocks for histological and immunohistochemical examination.

Lung tissue explants were obtained from the post-surgical material of 10 individuals with lung carcinoma who underwent lobectomy. The donors with primary lung carcinomas were used in this study. None of the individuals received treatment prior to surgery. Six specimens were obtained from NDRI (National Disease Research Interchange), four specimens were obtained in the Moscow City Clinical Hospital №62. The marginal part of the ectomized lung tissue was macroscopically intact, as assessed by the pathologist, and thus was excised for further tissue cultivation. The normal tissue morphology of the lung was additionally verified by histological examination of hematoxylin and eosin (H&E) tissue specimens on day 0 (tissue fixed on the day of surgery), thus the explants in the study corresponded to normal lung tissue.

The lung tissue of 6 individuals was used to set up the lung explant model, assess tissue viability and to analyze the immune cell content in cultured explants by flow cytometry.

The lung tissue of 4 individuals was used for SARS-CoV-2 infection experiments. The explant viability was monitored by lung tissue morphology on days 4, 7, and 10 of cultivation and compared to day 0. The viral infection was confirmed by qPCR and IHC. The cytokine concentrations were measured on beads using a commercial MILLIPLEX MAP Human kit (Merck Millipore).

All reagents used in the study are listed in **Supplementary Table S2**.

### 2.3 *Ex vivo* lung tissue

We delivered the lung tissue to the laboratory no later than 3 hours after surgery. We cut tissue under aseptic conditions into 2x2x2-mm blocks and randomly mixed them. Two blocks were fixed in 4% (v/v) formaldehyde (FA) for histological examination (day 0 of tissue culture). The rest of the tissue was cultured at the air–liquid interface on collagen rafts (Gelfoam, Pfizer, US). No fewer than 27 blocks of explants were placed into culture for each lung. The number of tissue blocks was 9 per 1/6 of gelfoam in a 6-well plate in culture medium containing RPMI-1640 (Gibco, Thermo Fisher Scientific, US), 15% (v/v) heat-inactivated FBS (HyClone, Cytiva, US); 1% (v/v) GlutaMAX (Gibco, Thermo Fisher Scientific); 1% (v/v) antibiotic/antimycotic (initial concentration 10,000 U/mL penicillin, 10,000 µg/mL streptomycin, 25 µg/ml amphotericin B, Gibco, Thermo Fisher Scientific); 1% (v/v) non-essential amino acids (Gibco, Thermo Fisher Scientific); 1% (v/v) sodium pyruvate (Gibco, Thermo Fisher Scientific). The tissue explants were cultured at 37°C in 5% CO_2_.

### 2.4 Flow cytometry of lung tissue cells

Lung explants of 6 individuals were collected at day 4-5 of cultivation and digested with 5mg/ml collagenase IV and 40U/ml DNase 1 (Thermo Fisher, US) for 30 minutes on a thermomixer at 37°C, 800 rpm. Cells were released using a pestle and filtered through 40 µm strainers along with cells retained from the collagen raft. Cells were washed and centrifuged at 500g for 5 minutes and stained with Live-Dead fixable stain (AlexaFluor 350, Thermo Fisher, US) for 20 minutes at room temperature, washed and centrifuged. Cells were treated with a 1:100 dilution of Fc Block (BD Biosciences, US) in BD stain buffer for 10 minutes at room temperature, washed and centrifuged. Cells were incubated for 30 minutes at room temperature with the following mouse anti-human antibodies in a total volume of 100µl per condition in BD stain buffer: CD45-APC-R700, CD3-BV510, CD4-BUV661, CD8-BUV395, CD11c-PE-Cy7, CD14-BUV805, CD16-BUV737, CD56-BUV496, CD66b-AlexaFluor 647, CD123-PE, and HLA-DR-APC-Cy7 (all antibodies were produced by BD Biosciences). Cells were washed, centrifuged and resuspended in 250µl of 4% paraformaldehyde for 1 hour. Data acquisition was performed using a FACSymphony A5 instrument with FACSDiva 9.3.1 acquisition software (BD Biosciences). Compensation controls were used to calculate fluorescence spillover, fluorescence minus one controls were used for gating and data was analyzed with FlowJo 10.8.1 (BD Biosciences). The gating strategy involved exclusion of debris, setting singlet and live cell gates, defining the CD45+ leucocyte population, defining gates for monocytes/macrophages based on CD14 and CD16, dendritic cells based on HLA-DR, CD11c and CD123, granulocytes based on CD66b, and setting a lymphocyte gate followed by gates for CD3+CD4+ T helpers, CD3+CD8+ cytotoxic T cells, and CD3-CD56+ natural killer cells.

### 2.5 SARS-CoV-2 stock and infection

All experiments using infectious SARS-CoV-2 were performed in a biosafety level 3 (BSL3) laboratory. We used SARS-CoV-2 B.1.1.1 variant (GISAID EPI_ISL_421275) for lung explant infection. The viral stock solution contained 10^7^ viral particles/mL as assessed from TCID50. The titration was performed on Vero E6 cell culture. The stock and its serial dilutions containing 10^6^ and 10^5^ particles/mL were used for infection.

After 24 h of cultivation, we replaced the culture medium to remove dead cells and cell debris, and infected the lung tissue with SARS-CoV-2 viral stock. We used serial stock dilutions of 10^5^, 10^6^, and 10^7^ viral particles/mL and added 10 μL of viral stock on top of each tissue block. Therefore, we added 10^3^, 10^4^, and 10^5^ viral particles on each lung explant. The number of blocks per collagen raft was 9; therefore, the total amount of viral particles in each well upon infection was 9x10^3^, 9x10^4^, or 9x10^5^. The tissue was incubated for 1 h at 37°C in 5% CO_2_, and the culture medium was changed to the fresh medium without the virus. In parallel with infected tissue, we cultured the non-infected controls. Every 3 days of culture (days 4, 7, and 10), we fixed 2 blocks of tissue with 4% FA for further histological examination and immunohistochemistry analysis and 2 blocks with RNAlater RNA stabilization reagent (Qiagen, the Netherlands) for qPCR, and we collected and replaced the conditioned culture medium. The collected medium was centrifuged at 3,000 rpm for 15 min, aliquoted, and stored at -80°C prior to measurement of viral RNA load and cytokine concentrations. The study design is presented in **Supplementary Figure S1**.

### 2.6 Histology and immunohistochemistry

The lung tissue specimens, 2x2x2 mm in size (obtained on days 0; 4; 7 and 10 of cultivation) were fixed in 4% FA for further histological and immunohistochemical (IHC) examination. Specimens were dehydrated in 50%, 70%, 100% ethanol and toluene according to the standard procedure, cast in paraffin blocks, and used to make 4-μm paraffin sections stained with hematoxylin and eosin (H&E). The staining was performed according to the manufacturer’s protocol; all dyes were provided by BioVitrum (Russia).

To confirm infection in cultured lung explants, we visualized SARS-CoV-2 N protein by immunohistochemistry on tissue sections. Anti-N protein antibodies (C706, rabbit monoclonal) were provided by HyTest (Finland) and used to detect SARS-CoV-2–infected cells. The preliminary step included incubation of the deparaffinized slides in 0.1% TritonX-100 for 1 h and blocking of the endogenous peroxidase using the Dual Endogenous Enzyme Block (Dako/Agilent, US) according to the manufacturer’s instructions. We performed the rest of the staining procedure using the UltraVision detection HRP DAB kit (Thermo, US) according to the manufacturer’s protocol. Additionally, anti-CD68 antibodies (mouse anti-human PG-M1; Talent Biomedical, China) were used to assess activated macrophages in the lung explants. The anti-CD68 staining was performed automatically using the Ventana BenchMark ULTRA system and reagents (Roche, Switzerland) according to the manufacturer’s instructions. The nuclei in all IHC specimens were additionally stained with hematoxylin.

The stained specimens were dehydrated and mounted in Shandon-Mount medium (Thermo Fisher Scientific, US). We imaged the sections using a Leica DM2000 microscope and a Leica DFC7000 T camera (Leica, Germany).

### 2.7 RNA extraction

For total RNA extraction from lung tissue, two 2x2x2-mm cultured lung explants per each time point were stored at -20°C in RNAlater RNA stabilization reagent (Qiagen, Germany). We mechanically disrupted the tissue from RNAlater using a FastPrep homogenizer and 0.56–0.7-mm garnet flakes (MP Biomedicals, US) in 700 μL of RLT buffer (Qiagen, Germany) supplemented with 1% β-mercaptoethanol (Merck, Germany). The homogenized specimens were centrifuged at 10,000 g and the supernatants were processed according to the RNeasy mini kit protocol (Qiagen, Germany). Total RNA was eluted in 100 μL of nuclease-free water and stored at -20°C for further viral load estimation.

For total RNA extraction from the conditioned culture media, we used the RIBO-prep kit (AmpliSens, Russia) according to the manufacturer’s protocol. Briefly, 100 μl of culture medium stored at -80°C was lysed, and nucleic acids were precipitated. The pellet obtained by centrifugation was washed and dissolved in 50 μL of nuclease-free water.

### 2.8 Reverse transcription qPCR

We detected SARS-CoV-2 RNA in the total RNA specimens of lung tissue and conditioned media using triple probe TaqMan real-time qPCR. We described the two sets of primers and probes for SARS-CoV-2 N2 and N3 detection previously (26) and used them simultaneously. We added the third primer/probe set for the *UBC* (ubiquitin C) gene as the internal normalization control. All primers and probes used for qPCR are listed in **Supplementary Table S3**.

The RNA solution (5 μL per well) was mixed with 5 μL of primer/probe mix and 10 μL of x2 OneTube RT-PCRmix (Sene, Russia). The PCR program was performed as follows: 20 min at 48°C for reverse transcription reaction followed by 5 min at 95°C, then 50 cycles, each comprising 20 s at 95°C, 20 s at 58°C, and 30 s at 72°C. All samples were analyzed in duplicates. For SARS-CoV-2 RNA copy number estimation, we generated the standard curve using 10-fold dilutions of synthetic DNA fragments containing N2 and N3 regions, as described previously (26).

### 2.9 Cytokine measurement

Forty-one cytokines in the culture medium were measured with a commercial kit MILLIPLEX MAP Human Cytokine/Chemokine Magnetic Bead Panel (Merсk Millipore). The cytokine panel included interleukin-1α (IL-1α), IL-1β, IL-1RA (IL-1 receptor antagonist), IL-2, IL-3, IL-4, IL-5, IL-6, IL-7, IL-8, IL-9, IL-10, IL-12 (p40), IL-12 (p70), IL-13, IL-15, IL-17A, fractalkine (CX3CL1), growth-regulated alpha (GRO-α or CXCL1), interferon-γ-induced protein-10 (IP-10 or CXCL10), monocyte chemoattractant protein-1 (MCP-1 or CCL2), MCP-3 (CCL7), macrophage inflammatory protein-1α (MIP-1α or CCL3), MIP-1β (CCL4), regulated on activation normally T-cell expressed and secreted (RANTES or CCL5), eotaxin (CCL11), macrophage-derived chemokine (MDC or CCL22), soluble CD40-ligand (sCD40L), epidermal growth factor (EGF), fibroblast growth factor-2 (FGF-2), Fms-like tyrosine kinase 3 ligand (Flt-3L), vascular endothelial growth factor (VEGF), granulocyte colony-stimulating factor (G-CSF), granulocyte-macrophage colony-stimulating factor (GM-CSF), platelet-derived growth factor-AA (PDGF-AA), PDGF-AB/BB, transforming growth factor-α (TGF-α), interferon-α2 (IFN-α2), IFN-γ, tumor necrosis factor-α (TNF-α), and TNF-β.

The standard curve was built up from 8 standard dilutions in triplicates, with 1–3 standard dilutions with dilution factor 5 and 4–8 dilutions with dilution factor 4. We used сulture medium to mimic the matrix effect on standard curves and blank wells. We used dilutions 1 and 1:50 with PBS (for cytokines with concentrations above the upper limit of detection).

Samples (25 μL) with dilutions 1 and 1:50, or standards and controls (25 μL), were diluted with 25 μL of assay buffer or with culture medium and incubated with 15 μL of 41-plex magnetic beads for 18 h at 4°C. Beads were washed twice manually with wash buffer on a magnet from the automatic magnetic washer ELx405 (Biotech) and incubated with detection antibodies for 1 h at 25°C. Antibodies were diluted with wash buffer 1.93 times and added in the amount 25 μL per well. After incubation, we added 15 μL of streptavidin–PE solution to the wells and incubated for 30 min at 25°C. Beads were washed twice, fixed with 1% FA for 1 h at room temperature, washed, and resuspended in wash buffer and analyzed on a Luminex 200 instrument. We collected 50 beads per region. During the analysis, we used a 5PL fit for the standard curve.

Steps prior to bead fixation were performed at the BSL3 laboratory. Cytokine measurement on the Luminex 200 instrument was performed at the BSL2 laboratory.

### 2.10 Statistical analysis

Statistical analysis was performed with R 4.2.1 software. The expression values obtained in the present study were in most cases not normally distributed, according to the Shapiro-Wilk test. For group comparison, we used the Wilcoxon signed-rank test with continuity correction. In order to overcome errors from multiple comparisons we performed a Benjamini-Hochberg FDR correction with calculation of critical values for each comparison matched with corresponding *p*-values; we calculated adjusted *p* values and compared them with a critical value of 0.05, if not stated otherwise.

For the correlation analysis we used Spearman’s correlation coefficients. The corresponding *p*-values with continuity correction for multiple comparisons were calculated. For calculation of Spearman’s coefficient, correlations with |R|≥0.5 and p. adj.≤0.05 were treated as significant.

We performed cytokine clusterization by the K-medoids clustering (27) method, which is more robust to noises and outliers, instead of k-means. Elbow and Silhouette methods used to define the optimal number of clusters.

## 3 Results

### 3.1 Development of the *ex vivo* lung tissue model

#### 3.1.1 Monitoring the lung tissue morphology using H&E sections

The normal morphology of intact lung tissue was confirmed by an experienced pathologist based on macroscopic evaluation and on H&E tissue sections analysis. A representative H&E stained lung tissue is provided in **Supplementary Figure S2**. The alveoli were not collapsed and retained normal morphology. The adjacent vessels contained a few erythrocytes and leucocytes. The cell nuclei remained intact, thus the non-stained part of the same lung specimen was considered normal and suitable for culturing.

To validate infected and non-infected lung explant viability, H&E staining was also employed. In the stained sections we assessed the collapsed vs. intact alveoli, tissue fibrosis and swelling, the presence of intact vs. lysed erythrocytes, cell karyolysis. The lung tissue morphology was mostly unaltered for up to day 7 of culture regardless of SARS-CoV-2 infection. We observed intact alveoli and cell nuclei (**Supplementary Figures S3A–D**). At day 10 we observed the collapse of the alveoli, in the specimens, the extracellular matrix was swollen and the erythrocytes were mostly lysed. However, the cells still contained intact nuclei with no karyolysis under all conditions (**Supplementary Figures S3E, F**). Thus, we concluded that the explants on the collagen rafts were generally viable up to day 10 of culture and thus were a suitable model for cytokine analysis.

#### 3.1.2 Monitoring the immune cell content and viability by flow cytometry

**Figure 1:**
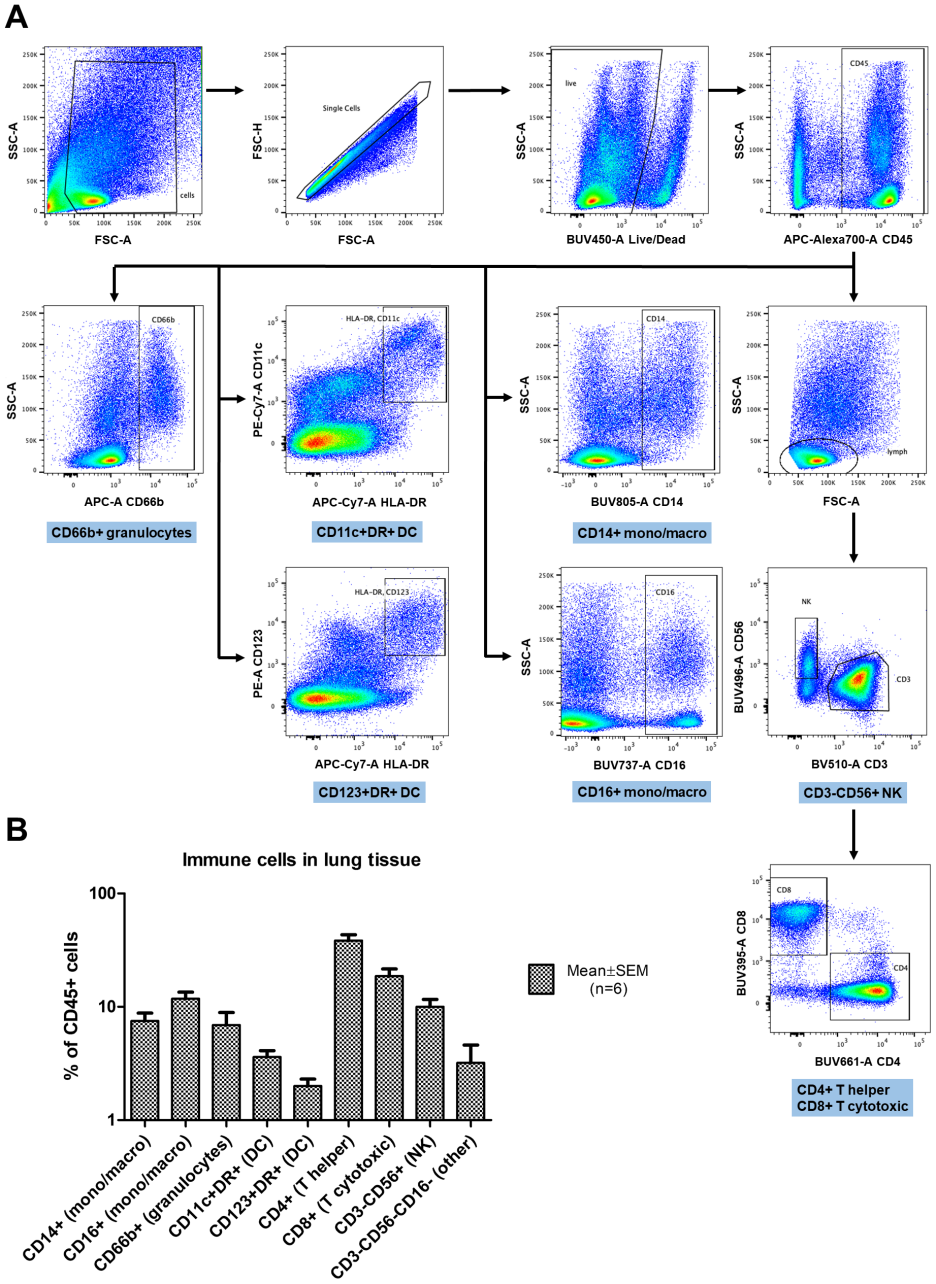
Flow cytometry analysis of immune cells in cultured lung explants enzymatically digested into cell suspensions. **(A)** The gating strategy: forward and side scatter (FSC, SSC) parameters were used to exclude debris and define single cell gates. Live cells were then identified and further limited to CD45+ leukocytes. Leukocyte subsets were defined based on expression of CD14 and CD16 (monocytes/macrophages); HLA-DR, CD11c, and CD123 (dendritic cells, DC); CD66b (granulocytes); CD3, CD4 and CD8 (T lymphocytes) or CD56 (natural killer cells, NK). **(B)** The percentages of leukocytes with specific subset markers in digested lung tissue (n=6), gated as shown in **(A)**. The data are presented as percentage of CD45+ cells (Mean ± SEM) in log scale.

*Ex vivo* lung tissue specimens of 6 individuals were analyzed for cell content and viability on day 4-5 of culture using flow cytometry. **Figure 1A** provides representative dot plots and the gating strategy for this analysis. The average amount of viable cells as evaluated by Live/Dead dye exclusion test on day 4-5 of culture was 81.8 ± 7.9% (Mean ± SEM). The average amount of CD45+ leucocytes recovered from digested lung explants and the underlying collagen raft, including both tissue-infiltrating cells and cells from the lung vessels was 47.5 ± 12.6% (Mean ± SEM). The average percentages of various leukocyte subpopulations are presented in **Figure 1B**. Thus the normal lung explants used for cultivation contained large amounts of viable diverse immune cells. The prevailing population of leukocytes were T helpers, followed by cytotoxic T cells. The intermediately represented populations were monocytes/macrophages and natural killer (NK) cells. The smallest populations observed were dendritic cells (DC) and granulocytes. The cytokine response provided by lung tissue with such immune cell content was further studied in SARS-CoV-2 infection experiments.

### 3.2 Immunohistochemical evaluation of infected lung tissue

**Figure 2:**
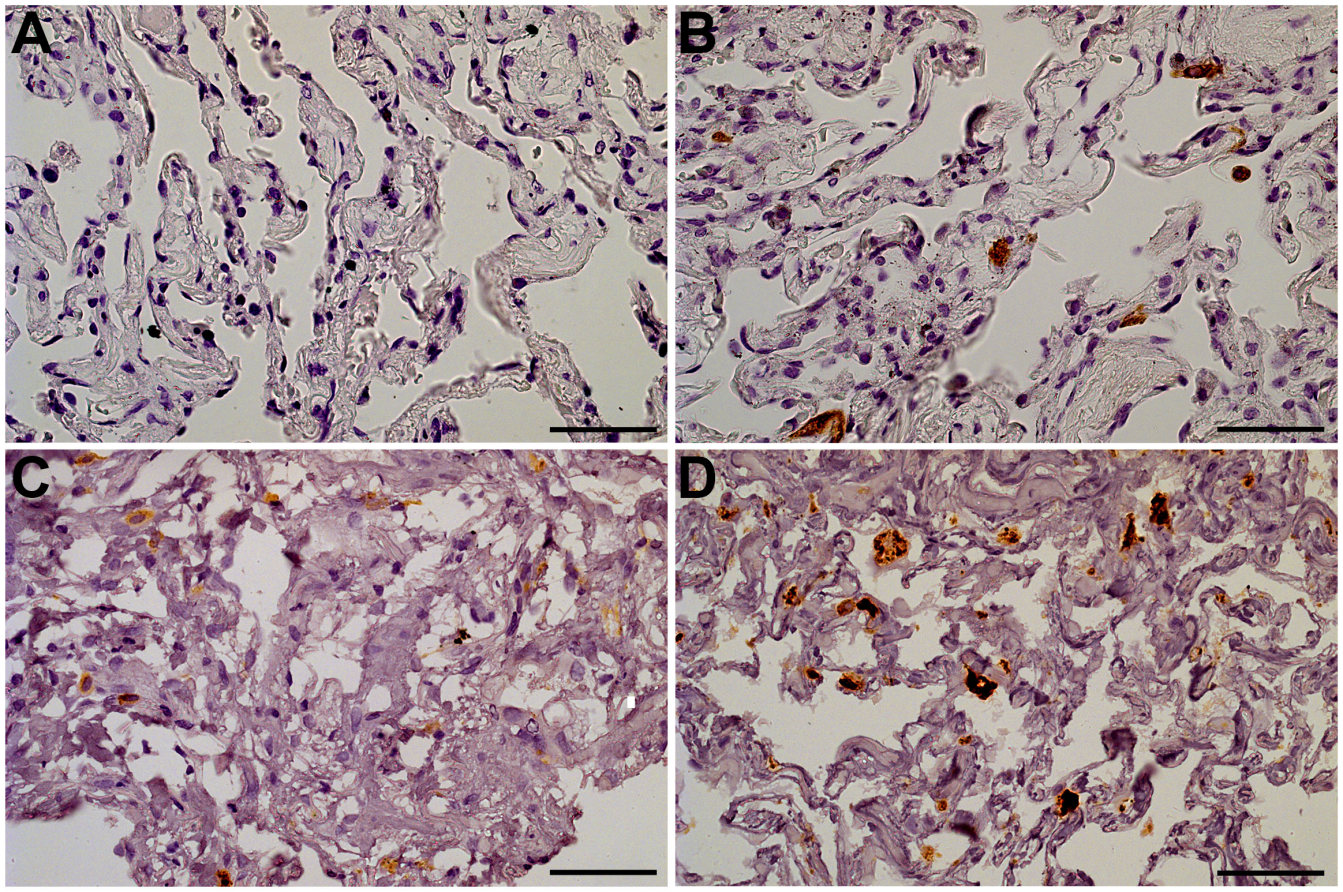
Lung tissue explants, day 4 of culture. The representative IHC stainings. **(A)** Anti-N-protein in a non-infected explant (specimen used as negative control); **(B)** Anti-N-protein in an explant infected with 10^7^ viral particles/mL (specimen contains single positive cells); **(C)** Anti-CD68 in an non-infected explant (macrophages exhibit CD68-positive staining); **(D)** Anti-CD68 in an explant infected with 10^7^ viral particles/mL (macrophages exhibit bright CD68-positive staining). Objective x40, scale bar 25 μm.

The staining of 18 autopsy lung specimens with SARS-CoV-2-associated pneumonia was used to assess the frequency of infected cells during lung infection *in vivo*. The infected cells that were positive for SARS-CoV-2 N-protein were typically single or assembled in small groups within pneumonia autopsy lungs (**Supplementary Figure S4**). Similarly, we observed single SARS-CoV-2-positive cells or small cell groups in explants infected *ex vivo* on day 4 of culture. The non-infected control explants demonstrated no positive staining (**Figures 2A, B**). The infected explants also showed brighter staining for CD68 than the non-infected control explants (**Figures 2C, D**), probably reflecting macrophage activation in infected tissues. Thus, we used IHC staining to confirm the infection in the lung explants. The characteristic pattern of infected cells in the explants was similar to that in the autopsy pneumonia. However, we were not able to detect SARS-CoV-2-positive cells in infected lung explants on days 7 and 10 of culture (**Supplementary Figure S5**).

### 3.3 SARS-CoV-2 RNA expression in lung *ex vivo* culture

**Figure 3:**
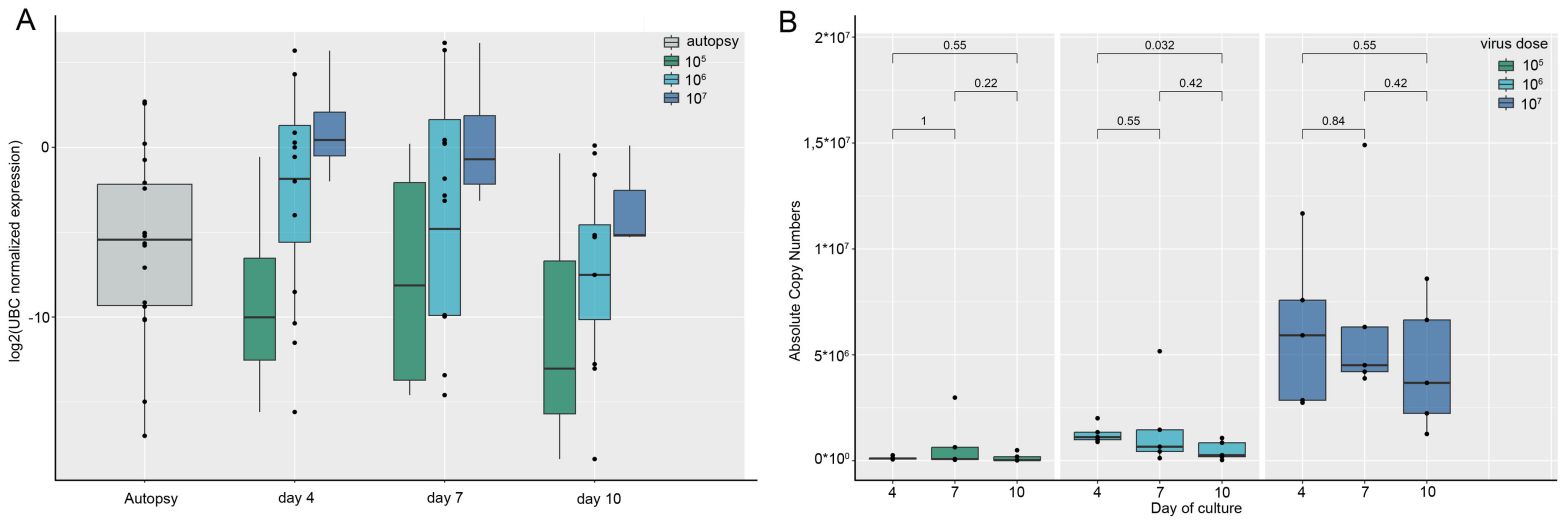
SARS-CoV-2 RNA expression in lung autopsy specimens compared to lung *ex vivo* culture. Presented are the results of individual experiments, medians and quartiles. **(A)** Tissue viral load. SARS-CoV-2 RNA tissue viral load normalized by the *UBC* reference gene in lung autopsy specimens and in lung tissue explants infected with SARS-CoV-2 during culture. The tissue viral load in the explants (except for 10^7^ particles/mL on day 4) does not differ significantly from the autopsies. *p* values are presented in **Supplementary Table S4**; **(B)** Viral load in conditioned culture media. The dynamics of SARS-CoV-2 viral load expressed in RNA absolute copy numbers in the conditioned culture media of lung explants infected with different concentrations of SARS-CoV-2 viral particles. SARS-CoV-2 copy numbers were calculated as an average between N2 and N3 copy numbers.

To assess the SARS-CoV-2 RNA levels associated with COVID-19 pneumonia patient autopsies were used. In the 18 reference COVID-19-associated pneumonia autopsies the estimated viral load normalized by the *UBC* (ubiquitin C) reference gene mRNA varied in a broad range: the median normalized viral load in the sample set amounted to 0.0233 [0.0016; 0.2232] (**Figure 3A**). With the unknown infection efficiency, a large range of initial virus concentrations was proposed in the infection model: it was decided to test 3 viral particle concentrations to infect lung tissue explants; thus, tissue explants were inoculated with 10^5^, 10^6^, and 10^7^ viral particles/mL. We then analyzed SARS-CoV-2 RNA content in tissue explants and in culture medium in dynamics. The *UBC*-normalized viral load in infected lung tissue explants fell in the same range as that in autopsy specimens (**Figure 3A**). *p* values for the comparisons of *UBC*-normalized viral load in autopsy specimens and tissue explants are presented in **Supplementary Table S4**.

The dynamics of viral RNA in the culture medium of infected tissue showed the presence of viral RNA during days 4, 7 and 10 of culture (**Figure 3B**). As viral load in tissue and culture medium in the infected lung *ex vivo* model was the highest after infection with 10^7^ viral particles/mL, we used the culture medium of tissue infected at this concentration for further analysis of cytokines.

### 3.4 Cytokine production in infected tissue

For the values falling below LLOD (lower limit of detection, the concentration of the maximally diluted standard) or exceeding the ULOD (upper limit of detection, the concentration of the minimally diluted standard), the following imputation techniques were employed. Initially, the quantity of missing values was estimated for each cytokine, as depicted in the **Supplementary Figure S6**. IL-3 had more than 40% missing and extrapolated values; therefore, we excluded it from the analysis. Conversely, the remaining cytokines had less than 40% missing or extrapolated values, rendering them eligible for further analysis. G-CSF, GRO-α, MCP-1, IL-6, and IL-8 were analyzed in dilution 1:50 because for these cytokines more than 40% of values were above the ULOD in dilution 1 and less than 40% of values were out of range in dilution 1:50. For the values below the LLOD, extrapolated values were used where available. Non-extrapolated cytokine concentrations below LLOD were replaced with the LLOD/2. Missed values above ULOD were replaced with ULOD.

**Figure 4:**
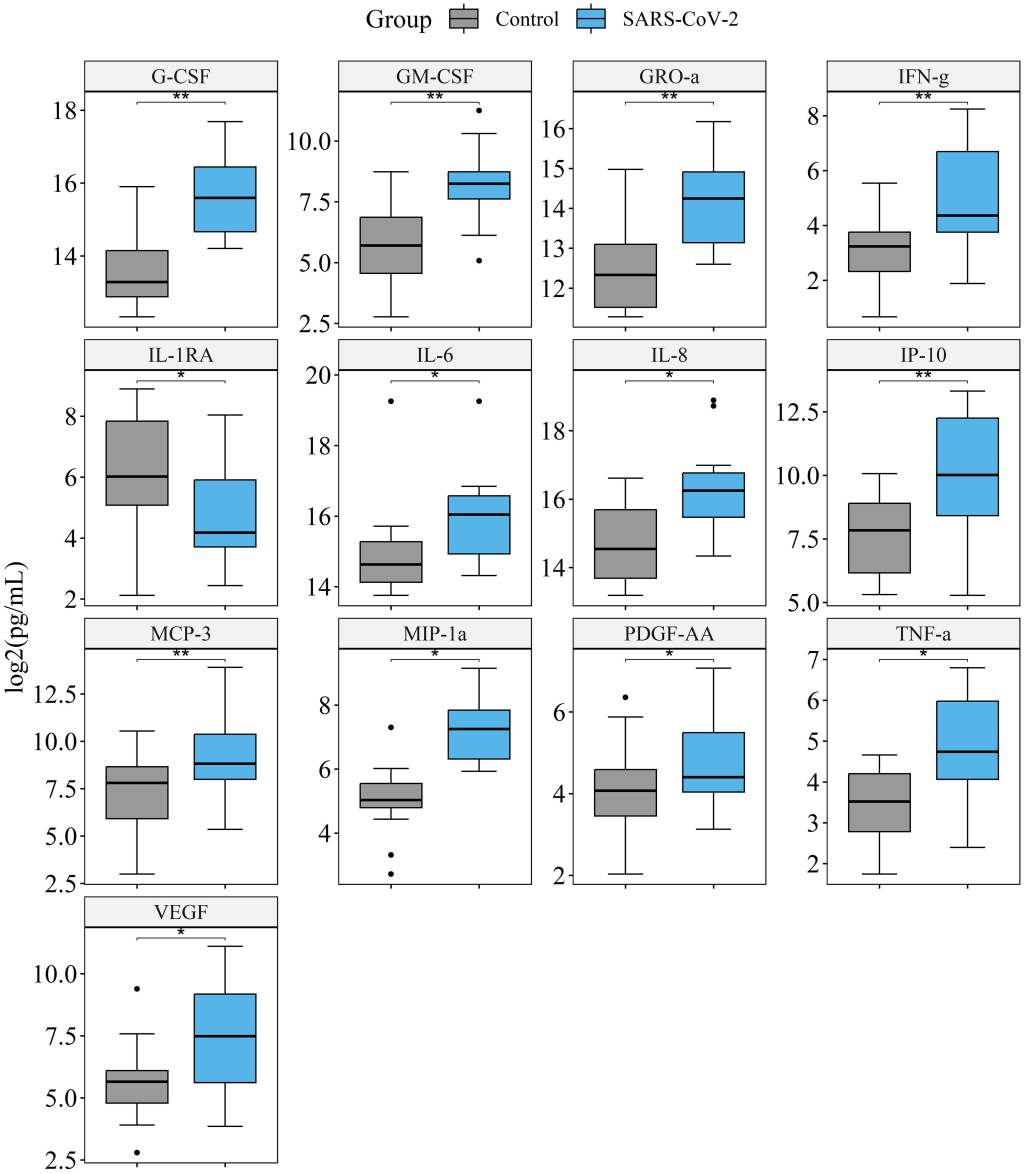
Comparison of cytokine production in the culture medium of lung tissue infected with 10^7^ SARS-CoV-2 viral particles/mL, and vehicle control. Presented are the cytokines that significantly differ in infected vs. non-infected tissue. The final sample set included 12 values for infected tissue and 12 values for non-infected tissue were analyzed for each cytokine. *p. adj.<0.05, **p. adj.<0.01, Wilcoxon signed-rank test.

We measured the concentrations of cytokines in the culture medium of lung tissue infected with 10^7^ SARS-CoV-2 particles per mL and in the vehicle control. Culture medium was replaced 1 h after infection and then collected and replaced on days 4, 7, and 10. Measurement of cytokines was performed for each time-point separately; then the results for days 4, 7, and 10 were included in one sample and analyzed with the Wilcoxon signed-rank test. The final sample included 24 values (12 for infected tissue, 12 for non-infected tissue) for each cytokine. In infected tissue compared with control tissue, we found an elevation of G-CSF, GM-CSF, GRO-α, IFN-γ, IL-6, IL-8, IP-10, MCP-3, MIP-1α, PDGF-AA, and VEGF concentrations, and a decrease of the IL-1RA concentration (**Figure 4**). Prior to *p* adjustment, IL-10, MIP-1β, and TNF-α were also elevated in infected tissue. However, with *p* adjustment these cytokines had a trend for elevation and did not achieve statistical significance (**Supplementary Table S5**). **Figure 4** shows cytokines differentially produced in infected vs. non-infected tissue. The results for all of the cytokines are presented in **Supplementary Table S5**. The dynamics of cytokine concentrations by time points during culture is presented in **Supplementary Table S6**.

### 3.5 Correlations of cytokines with viral load

**Figure 5:**
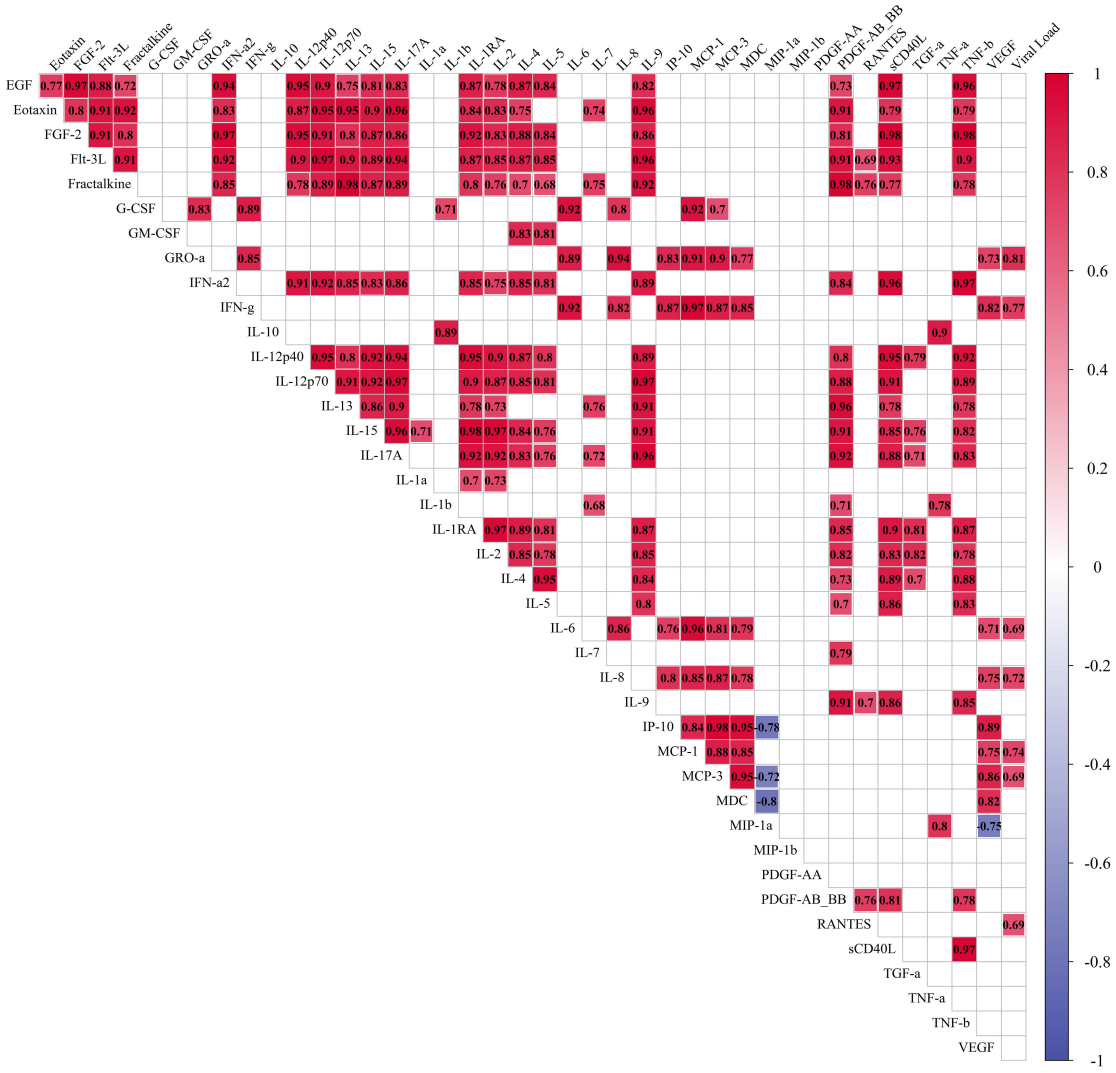
Correlations of cytokines in the culture medium of lung tissue infected with 10^7^ viral particles of SARS-CoV-2 per mL. Measurement of cytokines was performed for each time point separately, and then the results for days 4, 7, and 10 were included in one sample. The final sample set included 12 values for each cytokine. Red: correlations with Spearman’s correlation coefficient≥0.5, *p*. adj.≤0.05. Blue: correlations with Spearman’s correlation coefficient≤-0.5, *p*. adj.≤0.05. The size of the filling of each cell corresponds to the value of the Spearman’s correlation coefficient.

The correlation coefficients for cytokine levels for infected and non-infected tissue are provided in **Figure 5** and **Supplementary Figure S7** respectively. For infected tissue a correlation with viral load was also analyzed. We observed more positive correlations between cytokines in control (**Supplementary Figure S7**) than in infected tissue, which affected almost all measured cytokines. However, we found many new correlations (Spearman’s correlation coefficient≥0.5, *p*. adj.≤0.05) for IL-1RA, IL-5, IP-10, and MIP-1α with other cytokines in the culture medium of infected tissue (**Figure 5**). We found 7 cytokines which correlated with viral load, namely: GRO-α, IFN-γ, IL-6, IL-8, MCP-1, MCP-3, and RANTES (**Figure 5**).

### 3.6 Clusterization of cytokines

**Figure 6:**
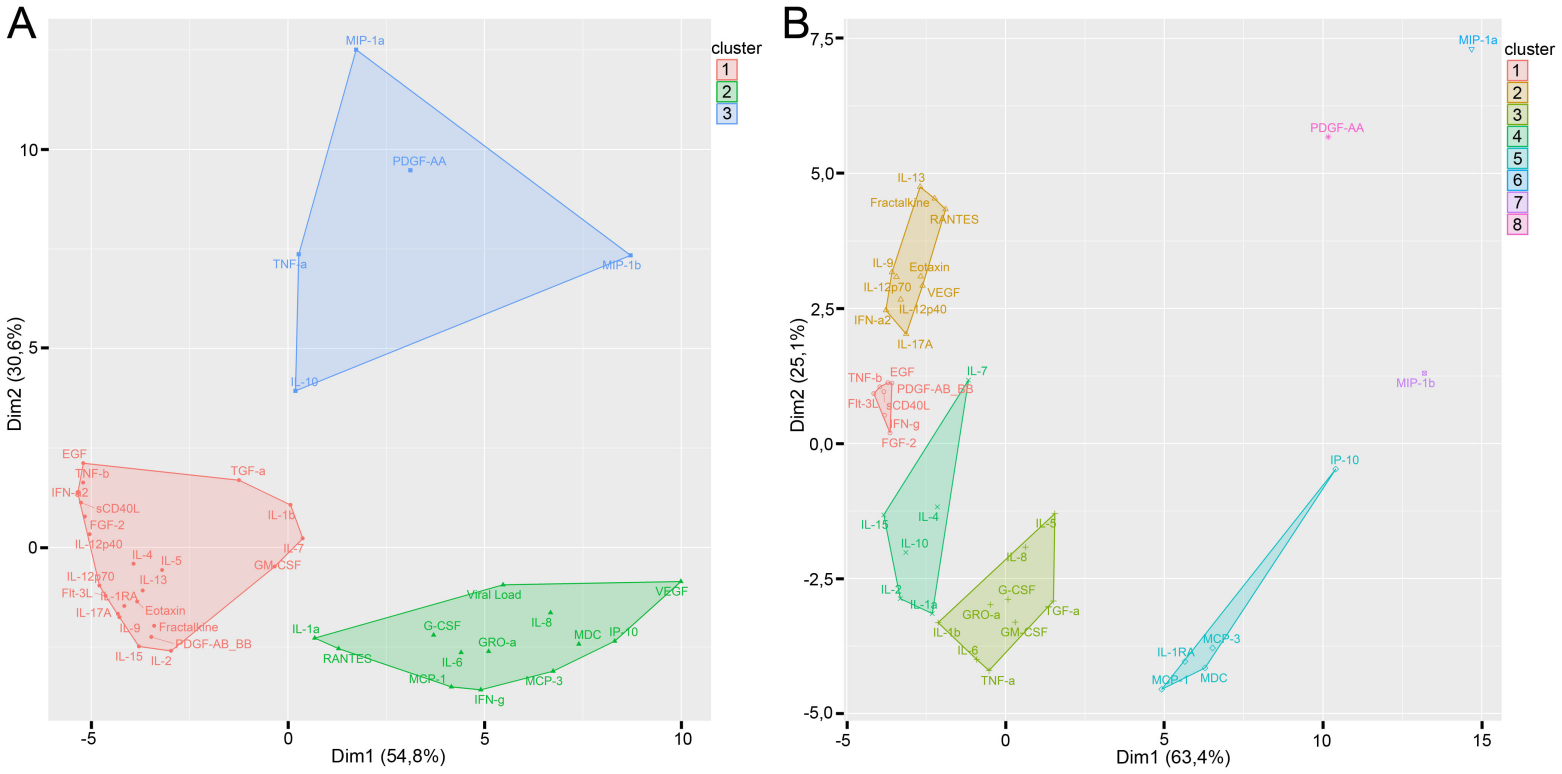
Clusterization of cytokines with K-medoids clustering method in infected tissue **(A)** and control tissue **(B)**. Optimal number of clusters was calculated by the Elbow and Silhouette methods.

We performed clusterization analysis of cytokines in infected (**Figure 6A**) and control tissue (**Figure 6B**) by K-medoids clustering method. The optimal number of clusters was calculated by Elbow and Silhouette methods.

We found three clusters of cytokines in infected tissues. Cluster 1 predominantly consisted of totally intercorrelated cytokines (**Figure 6A**). Cluster 1 contained only two cytokines that differed significantly in infected tissues and controls (GM-CSF and IL-1RA). Viral load and all of the cytokines which correlated with the viral load (GRO-α, IFN-γ, IL-6, IL-8, MCP-1, MCP-3, and RANTES) were included in cluster 2. Cytokines which differed between infected and control tissues were predominantly included in cluster 2 (G-CSF, GRO-α, IFN-γ, IL-6, IL-8, IP-10, MCP-3, and VEGF) and cluster 3 (MIP-1α, PDGF-AA, and trends for IL-10, MIP-1β, and TNF-α). Moreover, there were only three cytokines in clusters 2 and 3 which were not different in infected and non-infected tissues (IL-1α, IL-3, and MDC). Cytokines in cluster 3 had very few or no correlations.

Clusterization of cytokines in control tissues is shown in **Figure 6B**. Control tissues showed 5 clusters of cytokines; each cluster differed from clusters in infected tissues.

## 4 Discussion

Many studies on SARS-CoV-2 infection are performed in animal models and cell cultures. Moreover, novel techniques are used, including transwell models, lung-on-a-chip systems, and lung organoids (28–35). However, more sophisticated models are needed to further reproduce the structure and complexity of human lung tissue. To study the immune response to SARS-CoV-2 under laboratory-controlled conditions, we developed an *ex vivo* model of SARS-CoV-2 infection based on our previous studies with other tissues including tonsil, placental, and atherosclerotic plaque cultures (36–38).

In our lung *ex vivo* model, we demonstrated cell viability and the predominance of T cells by flow cytometry and by lung tissue morphology similarly to other authors (39, 40). We confirmed that until day 10 we did not observe the collapse of the alveoli, extracellular matrix swelling and erythrocyte lysis in the specimens. Moreover, the nuclei remained intact even on day 10 of culture. The previous estimates of tissue viability (39) showed viable lung cells in cultured explants until days 10-17 even after the collapse of the alveoli.

Here, we showed that SARS-CoV-2 infection in our model occurred well within the tissue viability limits: We observed N-protein-positive cells on day 4 of culture but did not find them on days 7 and 10. We attribute the lack of infected cells after day 7 to their limited number in a small tissue block. Thus, all available cells in the model were infected within a short range of time and therefore the primary immune *in situ* response could be observed without contribution of the systemic host inflammatory response. We found that SARS-CoV-2-infected cells were disseminated within the lung explant similarly to the autopsy specimens and the viral load in our experiments was in the same range as in the COVID-19 autopsies. Thus, our *ex vivo* lung model and the used virus titers reflect some important aspects of patients’ infection and allow to study *ex vivo* the initial stages of local cytokine response in the lung.

We hypothesized that since the architecture of the *ex vivo* lung tissue was largely preserved the cytokine profile in the SARS-CoV-2-infected model may correspond to the cytokine profile of infected lung tissue *in vivo*. To test this hypothesis, we measured the concentrations of 41 cytokines in the culture medium of infected and non-infected tissues using xMAP technology. We detected a significant elevation of G-CSF, GM-CSF, GRO-α, IFN-γ, IL-6, IL-8, IP-10, MCP-3, MIP-1α, PDGF-AA, and VEGF concentrations, and a reduction of the IL-1RA concentration in infected tissue. IL-10, MIP-1β, and TNF-α concentrations also tended to increase during SARS-CoV-2 infection. Most of the cytokines elevated in infected lung explants refer to pro-inflammatory response of innate and adaptive immunity and have been known to participate in lung inflammation of different types (41–54). Among the most prominent elevated cytokines we observed IL-6, a pleiotropic cytokine involved in host defense; its elevation may be a part of tissue response to viral infection and SARS-CoV-2-induced tissue injury (55). The elevation of IFN-γ may indicate the activation of antiviral response in the tissue. Interestingly, other authors who studied cytokines in lung explants reported interferon as missing in the anti-SARS-CoV-2 response (22, 56, 57) unlike the anti-H1N1 influenza or other coronaviruses. This apparent controversy may be due to the shorter culture period of the explants in these works. As our explants remained viable for 10 days, we were able to observe the cytokine response for longer and to detect the elevation of IFNγ in infected lung tissue.

Correlation and clusterization analyses revealed the cytokine pattern shift upon infection: three large clusters were formed upon SARS-CoV-2 infection, two of which contained the 11 upregulated cytokines. Moreover, we found GRO-α, IFN-γ, IL-6, IL-8, MCP-1, MCP-3, and RANTES to correlate with the viral load within a distinct cluster. These findings may indicate the cytokines in first line of involvement in SARS-CoV-2 infection.

We compared our results obtained in *ex vivo* culture with cytokine expression in COVID-19 patients in published studies and found similarity in cytokine profiles. G-CSF, GRO-α, IFN-γ, IL-6, IL-8, IP-10, and MIP-1α, elevated in our study, were previously shown to be elevated in the blood of patients with COVID-19 (58–61). Moreover, G-CSF, IFN-γ, IL-6, IP-10, MCP-3, and MIP-1α were shown to be associated with the severity of COVID-19 (58, 59, 62–64). In our previous study, G-CSF, GM-CSF, IL-6, IL-8, MCP-3, MIP-1α, and VEGF were elevated in the total group of COVID-19 patients with short-term combined clinical endpoint (transfer to intensive care, high-flow oxygen therapy, lung ventilation, and in-hospital mortality). Among these cytokines IL-6 and G-CSF exhibited the strongest difference between patients with and without the clinical endpoint. Moreover, IL-6 had the highest prognostic value for prediction of clinical endpoint in COVID-19 patients (65). Thus, a number of early stage cytokines detected in the lung tissue *ex vivo* are essentially similar to the cytokines that drive the systemic host response *in vivo*.

Taken together, our lung *ex vivo* explants retain their viability and support SARS-CoV-2 infection importantly reproducing the tissue viral load and infection dissemination through the tissue as well as the early stage cytokine profile characteristic of COVID-19 patients. As we have previously shown on other explanted tissues, such models are suitable for drug testing (66, 67) and co-infection studies (68–70). The developed system provides a laboratory-controlled model to investigate the mechanisms of lung infection by SARS-CoV-2 and by other viruses and potentially may be used for pre-clinical antiviral drug testing.

## Data Availability

The data that support the findings of this study are openly available at https://github.com/GeorgeRusakovich/Cytokine-production-in-ex-vivo-model-of-SARS-CoV-2-lung-infection.
