# Supplementary material for: Cytokine production in an ex vivo model of SARS-CoV-2 lung infection
Source: Front Immunol. 2024 Oct 21;15:1448515. doi: 10.3389/fimmu.2024.1448515 (PMC11532052; doi:10.3389/fimmu.2024.1448515)
Supplement: Supplementary file 1 [file DataSheet1.pdf]

Cytokine production in an *ex vivo* model of SARS-CoV-2 lung infection

Supplementary Material

1 Supplementary Figures and Tables

1.1 Supplementary Figures

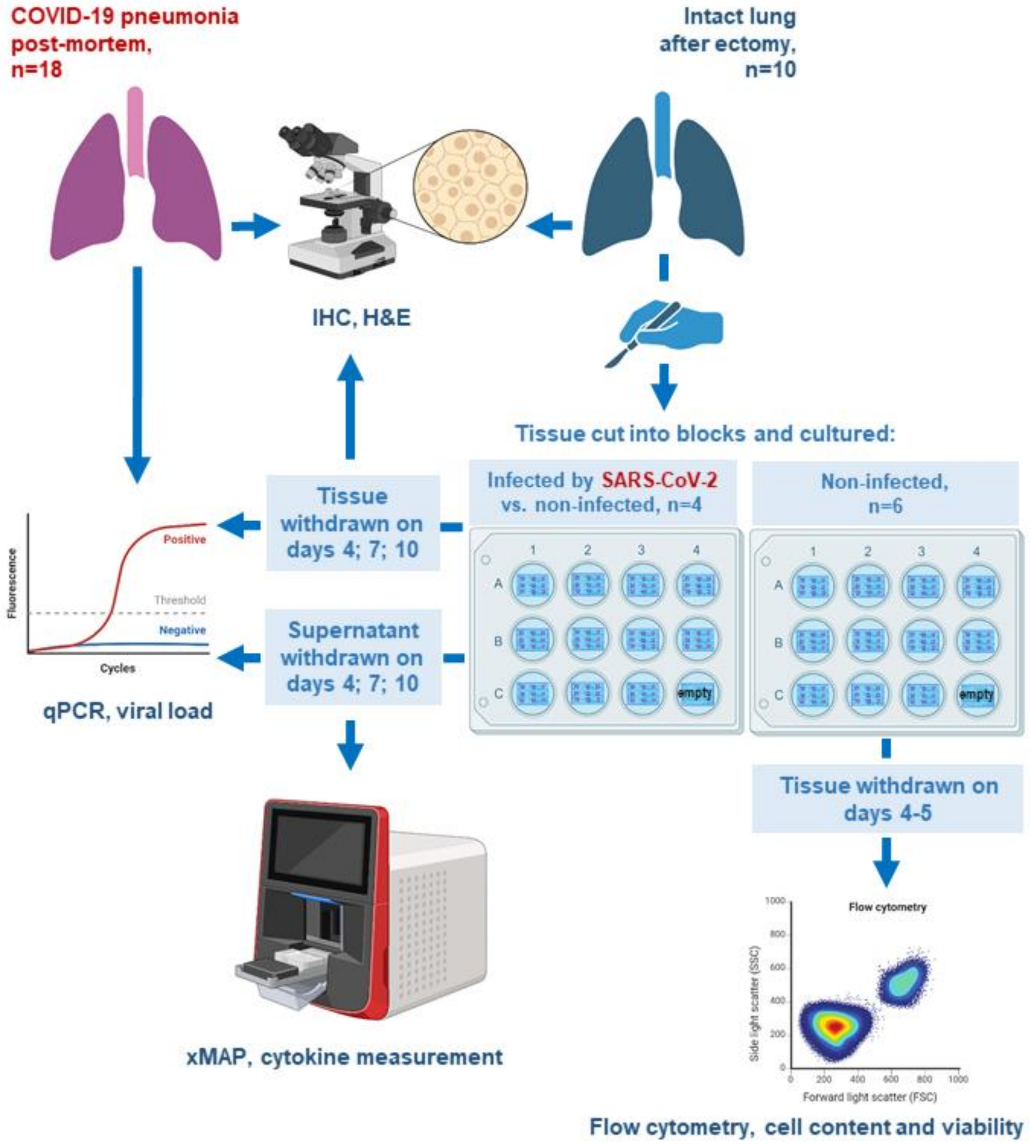

Supplementary Figure S1. Experimental setup. The lung specimens of post-mortem COVID-19 patients were used as reference to estimate the viral load in the tissue and to assess the anti-N-protein

staining in the infected lung. For lung tissue culture, the normal lung tissue was cut into 2x2x2 mm blocks and cultured at the air-liquid interface on collagen rafts at 37°C in 5% CO_2. One raft containing nine tissue blocks was placed into each well of a 12-well plate, one plate was used per patient. After 24 hours of culture, the culture medium was replaced to remove dead cells and cell debris. Tissue viability and immune cell content in the cultured explants were assessed by flow cytometry on days 4-5. For tissue infection, three concentrations of SARS-CoV-2 viral stock were used (10^5, 10^6 and 10^7 particles/mL). Infection was performed by placing 10 µl of viral stock on top of each lung explant. The tissue explants were incubated for 1 h at 37°C, then the culture medium was changed to the fresh medium without the virus. The non-infected control explants were cultured along with infected tissue. Every 3 days of culture (days 4, 7 and 10) 2 blocks of tissue were placed in 4% formaldehyde for further histological (H&E) and IHC analysis, 2 blocks were placed in RNA stabilization reagent for tissue viral load estimation by qPCR. The culture medium was also collected and replaced with fresh medium on days 4, 7 and 10 and used for viral load estimation and cytokine secretion analysis using xMAP technology. The scheme of the experimental setup was generated using BioRender – www.biorender.com

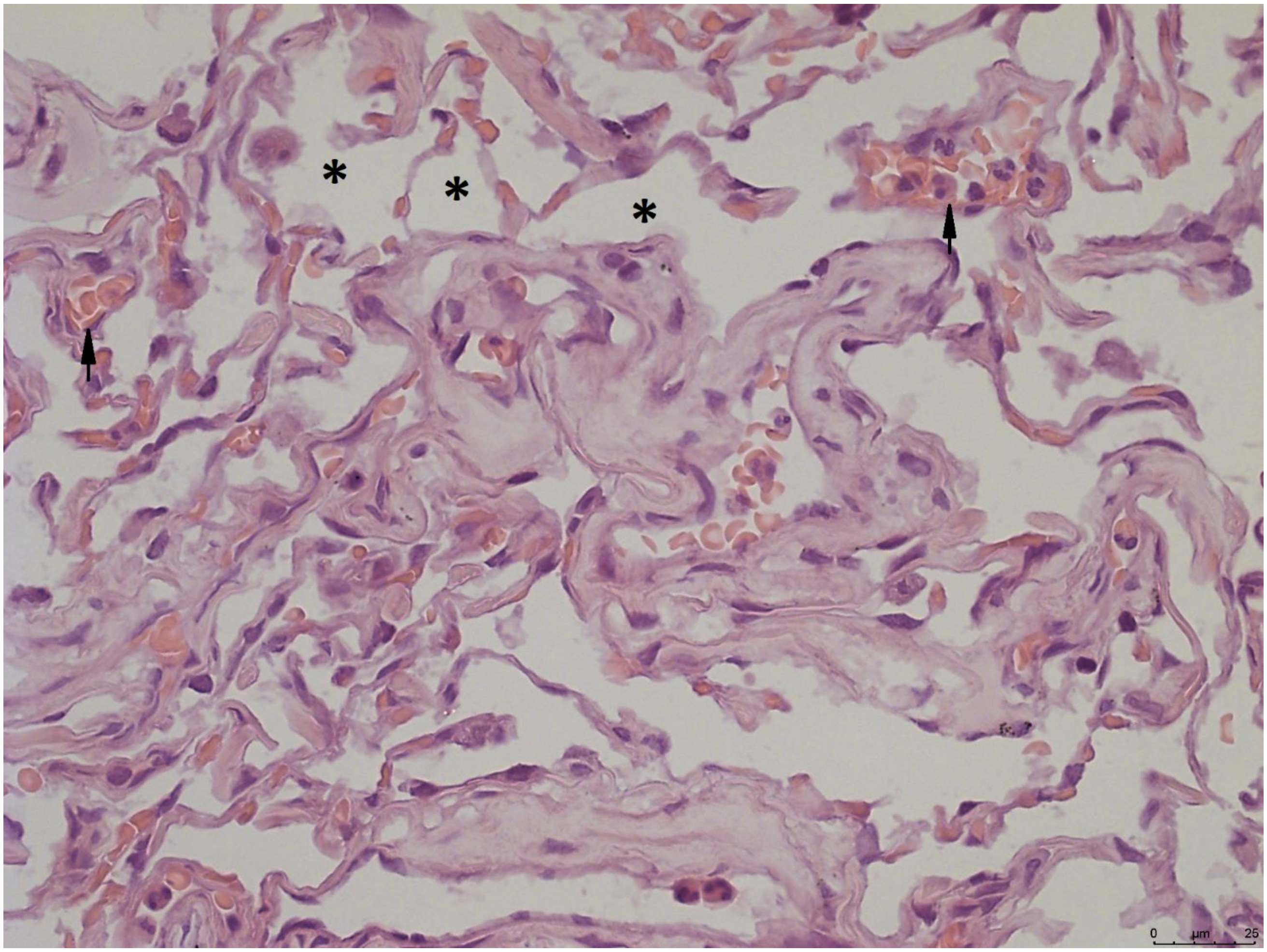

Supplementary Figure S2. Lung tissue morphology directly after surgery - a macroscopically intact area was excised for future cultivation (day 0 of explant cultivation). The alveoli (marked by black asterisks) are intact with thin walls, the vessels (marked by black arrows) contain intact erythrocytes and leukocytes. H&E staining. Objective x40, scale bar 25 µm.

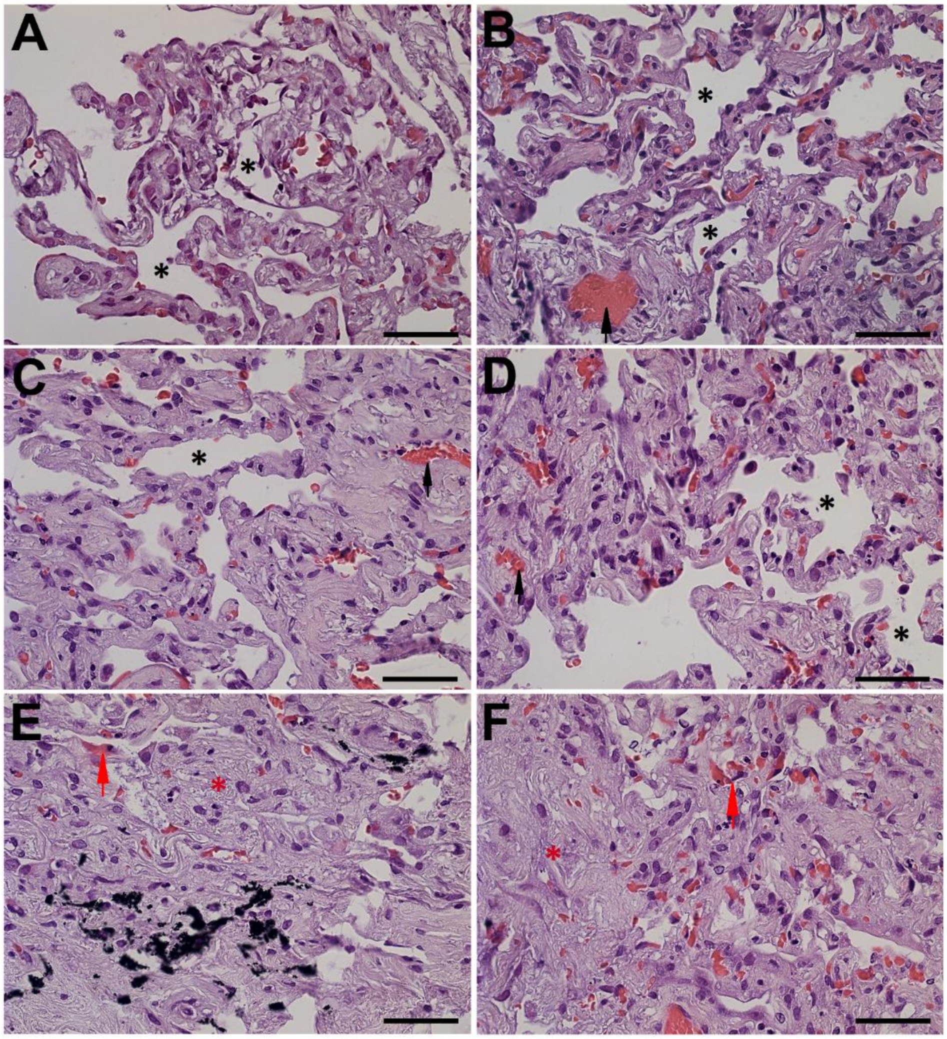

Supplementary Figure S3. Lung tissue upon explant culture - monitoring tissue morphology. H&E staining. (A) A non-infected explant, day 4 of culture. The lung morphology is unchanged; the alveoli (marked by black asterisks) are intact; the cell nuclei show no signs of karyolysis. (B) An explant infected with 10^7 viral particles/mL, day 4 of culture. The lung morphology is unchanged; the alveoli (marked by black asterisks) are intact; the vessel (marked by black arrow) contains intact erythrocytes; the cell nuclei show no signs of karyolysis. (C) A non-infected explant, day 7 of culture. The lung morphology is unchanged; the alveoli (marked by black asterisk) are intact; the vessel (marked by black arrow) contains intact erythrocytes; the cell nuclei show no signs of karyolysis. (D) An explant infected with 10^7 viral particles/mL, day 7 of culture. The lung morphology is unchanged; the alveoli (marked by black asterisks) are intact; the vessel (marked by black arrow) contains intact erythrocytes; the cell nuclei show no signs of karyolysis. (E) A non-infected explant, day 10 of culture. The alveoli are collapsed and the extracellular matrix is swollen (marked by red asterisk); the erythrocytes show signs of hemolysis (marked by red arrow); the cell nuclei show no signs of karyolysis. (F) An explant infected with 10^7 viral particles/mL, day 10 of culture. The alveoli are collapsed and the extracellular matrix is swollen (marked by red asterisk); the erythrocytes show signs of hemolysis (marked by red arrow); the cell nuclei show no signs of karyolysis. Objective x40, scale bar 25 µm.

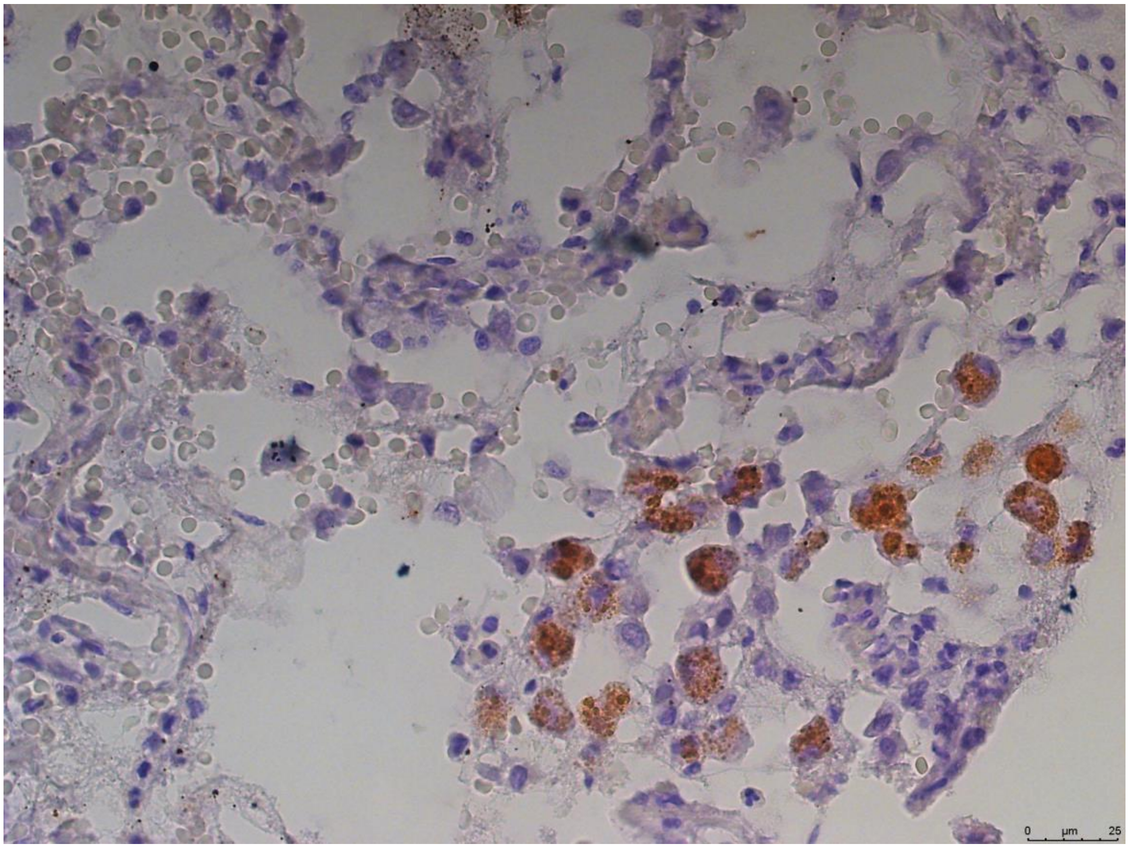

Supplementary Figure S4. A representative autopsy lung with SARS-CoV-2-associated pneumonia. Anti-SARS-CoV-2 N-protein staining, IHC, HRP+hematoxylin. The rounded cells with uneven cytoplasm and bright positive anti-SARS-CoV-2 staining are located in a small group within the lung. Objective x40, scale bar 25 μm.

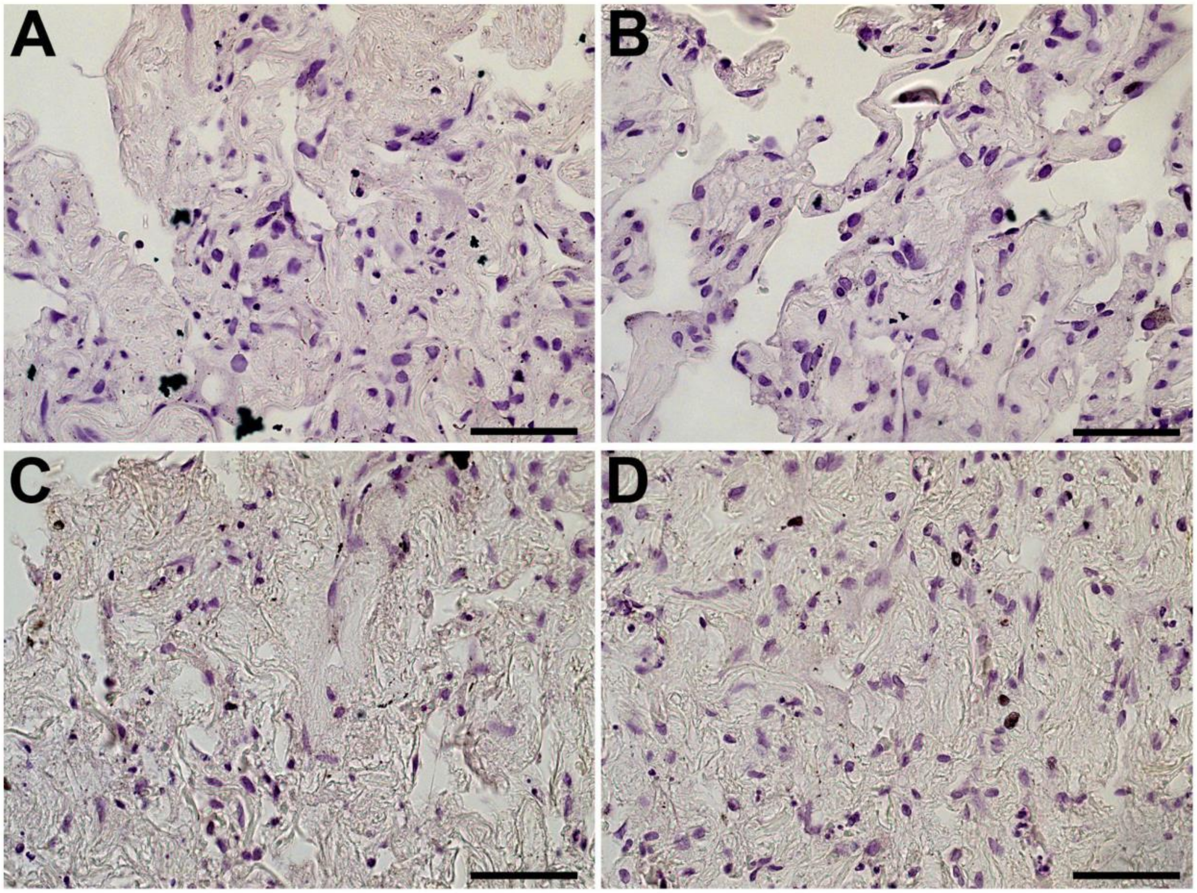

Supplementary Figure S5. A representative anti-SARS-CoV-2 N-protein staining in lung explants after prolonged cultivation. (A) A non-infected control explant, day 7 of culture. (B) An explant infected with 10^7 viral particles/mL, day 7 of culture. (C) A non-infected control explant, day 10 of culture. (D) An explant infected with 10^7 viral particles/mL, day 10 of culture. No infected cells were detected. IHC, HRP+hematoxylin. Objective x40, scale bar 25 μm.

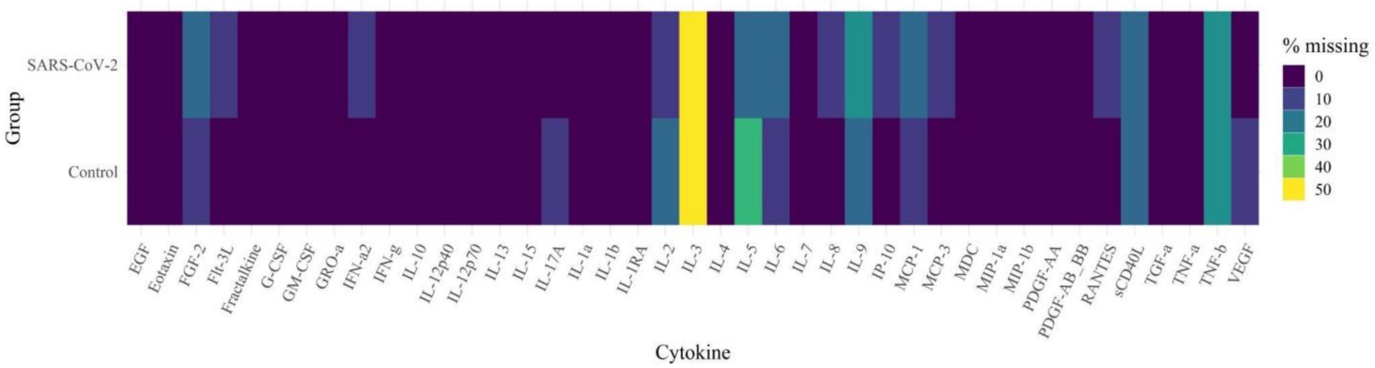

Supplementary Figure S6. The percentage of missing and extrapolated values for each cytokine in the culture medium. SARS-CoV-2 refers to the explants infected with 10^7 viral particles/mL; Control refers to non-infected explants. 41 cytokines were measured using xMAP technology. For G-CSF, GRO-a, MCP-1, IL-6, IL-8, dilution 1:50 was used. For other cytokines we used dilution 1.

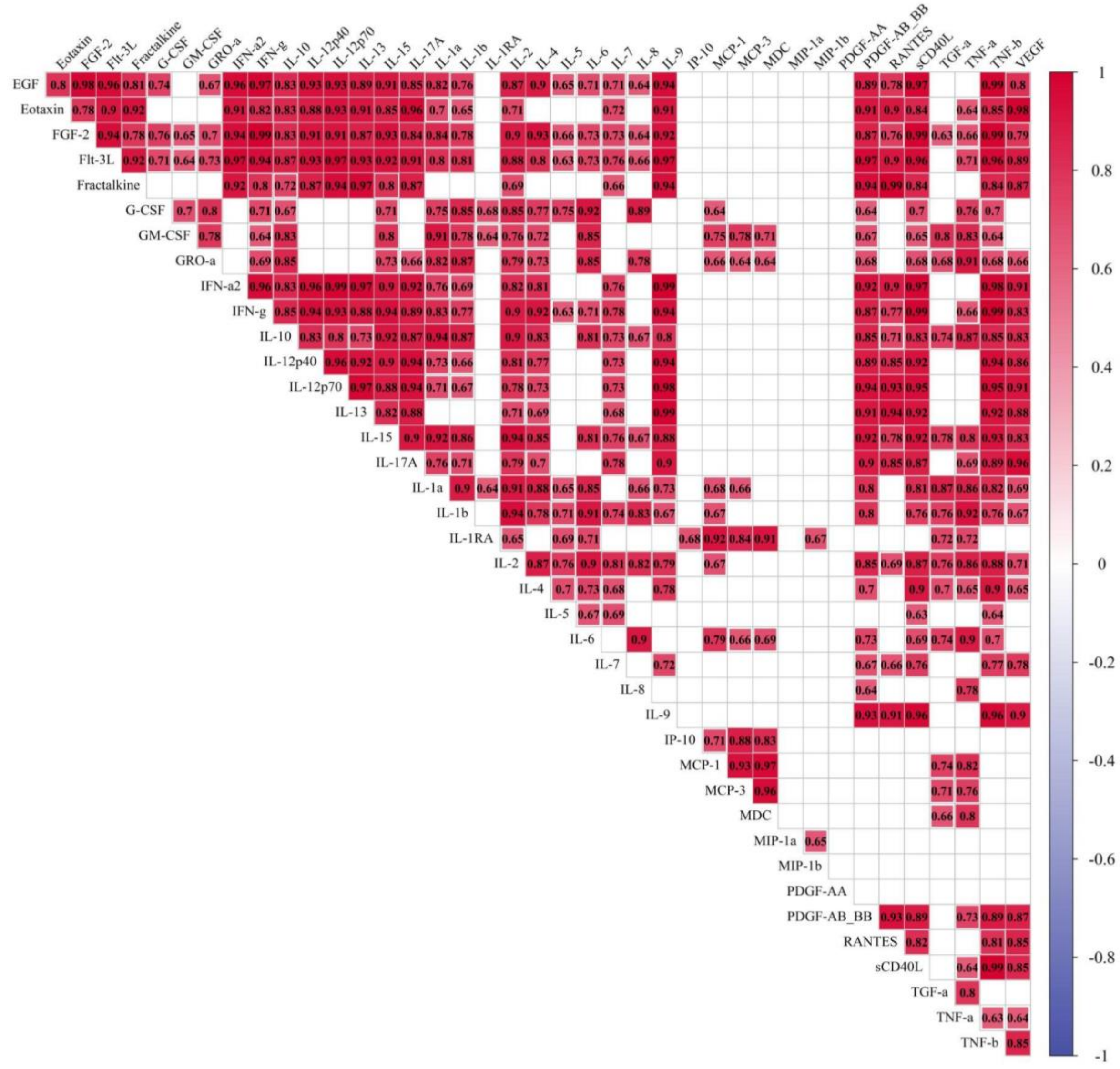

Supplementary Figure S7. Correlations of cytokines in the culture medium of the non-infected lung tissue. Measurement of cytokines was performed for each time point separately, then the results for days 4, 7 and 10 were included in one sample. The final sample set included 12 values for each cytokine. Red - correlations with Spearman's correlation coefficient>=0.5, p adj<0.05. Blue - correlations with Spearman's correlation coefficient<=-0.5, p adj<0.05. The size of the filling of each cell corresponds to the value of the Spearman's correlation coefficient.

1.2 Supplementary Tables

Supplementary Table S1. Clinical characteristics of autopsy pneumonia specimens with COVID-19 as a cause of death. The specimens were used as reference for tissue viral load estimation and IHC visualization of infected cells.

Patient #	Sex	Age	Lung CT on admission	Lung CT in hospital	SARS-CoV-2 RNA in lung tissue normalized by *UBC**
1	female	82	NA	NA	6.48071580
2	male	79	3	NA	6.00735955
3	female	84	2	2	1.16157195
4	male	79	2	3	0.59922795
5	female	78	3	NA	0.23558830
6	male	84	NA	NA	0.18591630
7	female	NA	3	4	0.03024870
8	male	73	3	3	0.02976095
9	male	65	NA	NA	0.02681640
10	male	73	3	4	0.01819065
11	female	82	NA	NA	0.01976185

12	male	NA	4	NA	0.00732370
13	male	79	3	3	0.00149725
14	female	68	NA	NA	0.00086520
15	male	62	3	4	0.00176320
16	female	68	NA	NA	0.00089429
17	female	61	3	3	0.00003072
18	male	89	2	3	0.00000761

*SARS-CoV-2 RNA viral load was calculated as the average copy number of N2 and N3 regions. CT - computed tomography. *UBC* - ubiquitin C gene. NA - not available.

Supplementary Table S2. The reagents used in the study.

№	Reagent	cat #	Manufacturer	Country of origin
1	Vero E6	CRL-1586	ATCC	US
2	Formaldehyde (FA)	158127	Sigma-Aldrich/Merck Life Science LLC	Germany
3	Gelfoam, Pfizer	00009031508	Gelfoam ®/Pfizer	US
5	RPMI-1640	11875093	Gibco™/Thermo Fisher Scientific	US
6	FBS HyClone	SH30071.03	HyClone™/Cytiva	US
7	GlutaMAX	35050087	Gibco™/Thermo Fisher Scientific	US
8	Non-essential amino acids	11140050	Gibco™/Thermo Fisher Scientific	US
9	Sodium pyruvate	11360070	Gibco™/Thermo Fisher Scientific	US
10	RNAlater (RNA stabilization reagent)	AM7020	Invitrogen™/Thermo Fisher Scientific	The Netherlands
11	Ethanol	100967	Millipore/Merck Life Science LLC	Germany
12	Toluene	589578100	Sigma-Aldrich/Merck Life Science LLC	Germany
13	Paraffin HISTOMIX	247/NS	BioVitrum	Russia
14	Eosin solution	HK-ES-B250	BioVitrum	Russia
15	Hematoxylin solution	HK-G0-CD05	BioVitrum	Russia
16	Anti-N protein antibodies	Gift: C706, rabbit monoclonal	HyTest	Finland
17	0.1% TritonX-100	X100-100ML	Sigma-Aldrich/Merck Life Science LLC	Germany
18	Dual Endogenous Enzyme Block	S200380-2	Dako/Agilent	US
19	UltraVision detection HRP DAB kit	TL-060-QHD	LabVision Corp./Thermo Fisher Scientific	US
20	Anti-CD68 antibodies	AR0349	Talent Biomedical	China
21	Shandon-Mount medium	1900333	Epredia™ /Thermo Fisher Scientific	US
22	RLT buffer	79216	QIAGEN	Germany

23	β-mercaptoethanol	444203	Calbiochem®/Merck Life Science LLC	Germany
24	RNeasy mini kit protocol	74104	QIAGEN	Germany
25	RIBO-prep kit	K2-9-Et-100	AmpliSens®	Russia
26	OneTube RT-PCRmix	SK101M	Eurogene	Russia
27	MILLIPLEX MAP Human Cytokine/Chemokine Magnetic Bead Panel	HCYTMAG-60K-PX41	Millipore/Merck Life Science LLC	Germany
28	Collagenase, Type IV, powder	17104019	Gibco™/Thermo Fisher Scientific	US
29	DNase I	18047019	Invitrogen™/Thermo Fisher Scientific	US
30	Live-Dead fixable stain AlexaFluor 350	L23105	Invitrogen™/Thermo Fisher Scientific	US
31	Human BD Fc Block™	564219	BD Pharmingen™	US
32	Stain Buffer (FBS)	554656	BD Pharmingen™	US
33	APC-R700 Mouse Anti-Human CD45	566041	BD Pharmingen™	US
34	BD Horizon™ BV510 Mouse Anti-Human CD3	566779	BD Pharmingen™	US
35	BD Horizon™ BUV661 Mouse Anti-Human CD4	569782	BD Pharmingen™	US
36	BD Horizon™ BUV395 Mouse Anti-Human CD8	569178	BD Pharmingen™	US
37	BD™ PE-Cy™7 Mouse Anti-Human CD11c	652358	BD Pharmingen™	US
38	BD Horizon™ BUV805 Mouse Anti-Human CD14	612902	BD Pharmingen™	US
39	BD Horizon™ BUV737 Mouse Anti-Human CD16	612786	BD Pharmingen™	US
40	BD Horizon™ BUV496 Mouse Anti-Human NCAM-1 (CD56)	569467	BD Pharmingen™	US
41	Alexa Fluor® 647 Mouse Anti-Human CD66b	561645	BD Pharmingen™	US
42	BD™ PE Mouse Anti-Human CD123	340545	BD Pharmingen™	US

43	BD™ APC-Cy™7 Mouse Anti-Human HLA-DR	335796	BD Pharmingen™	US

Supplementary Table S3. Primers and TaqMan probes for SARS-CoV-2 detection in lung tissue.

Name	Sequence	Label
N2-forward	TTACAAACATTGGCCGCAAA	-
N2-reverse	GCGCGACATTCCGAAGAA	-
N2-probe	ACAATTTGCCCCCAGCGCTTCAG	5’-FAM 3’-BHQ1
N3-forward	GGGAGCCTTGAATACACCAAAA	-
N3-reverse	TGTAGCACGATTGCAGCATTG	-
N3-probe	ATCACATTGGCACCCGCAATCCTG	5’-VIC 3’-BHQ2
UBC-forward	TTGGGTCGCAGTTCTTGTTTG	-
UBC-reverse	TGCCTTGACATTCTCGATGGT	-
UBC-probe	TCGCTGTGATCGTCACTTGACAATG	5’-ROX 3’-BHQ2

*the *N2* and *N3* SARS-CoV-2 sequences used were recommended for SARS-CoV-2 (Accession ID MN908947.3) detection by Division of Viral Diseases, National Center for Immunization and Respiratory Diseases, Centers for Disease Control and Prevention, Atlanta, GA, USA. *UBC* (Accession ID NM_021009) was added as a reference gene. All oligonucleotides were synthesized by DNK-Sintez (Russia) and put into a triplex one-step qPCR reaction. Sequences are presented in 5′-3′ direction. FAM – fluorescein; VIC – 2′-chloro-7′phenyl-1,4-dichloro-6-carboxy-fluorescein; ROX – carboxyrhodamine; BHQ1/2 – Black Hole Quencher 1/2.

Supplementary Table S4. *p* values for the comparisons of *UBC*-normalized SARS-CoV-2 viral load in autopsy specimens and lung explants.

Group	10^5-Culture day 4	10^5-Culture day 7	10^6-Culture day 10	10^6-Culture day 4	10^6-Culture day 7	10^7-Culture day 10	10^7-Culture day 4	10^7-Culture day 7	Autopsy
**10^5-Culture day 10**	0.857	0.857	0.700	0.229	0.229	0.400	0.114	0.229	0.356
**10^5-Culture day 4**		0.886	0.857	0.200	0.343	0.229	0.057	0.114	0.342
**10^5-Culture day 7**			0.857	0.343	0.343	0.857	0.114	0.200	0.712
**10^6-Culture day 10**				0.400	0.629	0.400	0.114	0.229	0.669
**10^6-Culture day 4**					1.000	0.629	0.486	0.486	0.342
**10^6-Culture day 7**						1.000	0.686	0.486	0.652
**10^7-Culture day 10**							0.229	0.229	0.534
**10^7-Culture day 4**								0.886	0.019
**10^7-Culture day 7**									0.042

p values were not adjusted for multiple comparisons. Green - $p<0.05$.

Supplementary Table S5. Cytokine concentrations in lung tissue explants infected with SARS-CoV-2.

Cytokine	Control	SARS-CoV-2	*p*	*p*. adj.
EGF	10.49 [5.4825; 14.7525]	10.97 [7.1275; 13.7625]	0.51855	0.72
Eotaxin	13.83 [10.14; 20.4675]	13.17 [11.3625; 15.78]	0.96973	0.97
FGF-2	41.33 [25.93375; 61.0475]	49.5 [25.065; 55.6525]	0.62207	0.78
Flt-3L	21.395 [9.3; 29.6025]	16.86 [13.3375; 26.02]	0.90967	0.96
Fractalkine	166.715 [99.1525; 306.3475]	152.02 [86.99; 277.7075]	0.90967	0.96
G-CSF	10033.635 [7535.91; 18221.4725]	49396.77 [25992.9375; 89048.9025]	0.00049	0.0039
GM-CSF	57.73 [23.53; 116.46]	304.34 [198.5475; 442.315]	0.00049	0.0039
GRO-α	5178.34 [2937.06; 8789.1075]	19449.285 [9034.865; 30918.6575]	0.00049	0.0039
IFN-α2	32 [19.855; 45.55]	31.24 [20.51625; 36.625]	0.67725	0.8
IFN-γ	9.405 [5.035; 13.51]	20.635 [13.7025; 108.4425]	0.00049	0.0039
IL-10	13.015 [5.81; 21.2075]	28.82 [20.6425; 48.2875]	0.02686	0.077

IL-12p40	20.785 [11.4125; 34.575]	22.36 [16.73; 27.7825]	0.51855	0.72
IL-12p70	9.33 [4.4175; 18.1375]	7.615 [5.7375; 11.41]	0.96973	0.97
IL-13	12.635 [7.4425; 22.035]	10.945 [8.3625; 16.78]	0.85010	0.94
IL-15	10.325 [6.2975; 14.22]	8.81 [5.625; 11.68]	0.62207	0.78
IL-17A	4.595 [2.0975; 7.3525]	5.535 [2.8325; 9.8775]	0.30127	0.45
IL-1α	7.185 [3.7825; 11.6375]	9.56 [7.9475; 12.14]	0.06396	0.13
IL-1β	17.335 [15.8825; 20.595]	19.335 [17.6425; 25.2325]	0.05225	0.12
IL-1RA	66.705 [33.8525; 228.745]	18.14 [13.8475; 60.1425]	0.01221	0.041
IL-2	2.67 [0.785; 3.295]	2.235 [1.565; 3.2025]	0.26640	0.41
IL-4	137.965 [37.0825; 169.03]	147.915 [126.5175; 172.435]	0.06396	0.13
IL-5	1.835 [0.335; 4.0125]	2.3 [0.935; 5.305]	0.19733	0.34
IL-6	25843.73 [17954.6175; 39615.5475]	67617.225 [31339.345; 98138.095]	0.00386	0.022
IL-7	67.255 [64.05; 77.2125]	69.905 [66.8275; 73.1575]	0.23340	0.39
IL-8	24435.215 [13208.51;	77820.365 [46126.3575;	0.00488	0.022

	53378.3525]	111793.7325]		
IL-9	2.815 [1.2975; 6.465]	3.64 [1.5275; 5.5275]	0.26611	0.41
IP-10	231.39 [73.275; 486.1925]	1034.175 [365.0975; 5119.6825]	0.00146	0.0098
MCP-1	54747.51 [23636.9775; 102414.1]	75546.685 [25526.5425; 141987.8025]	0.06835	0.13
MCP-3	231.185 [60.9925; 405.1975]	456.055 [254.335; 1339.9925]	0.00049	0.0039
MDC	174.1 [65.1625; 457.7125]	299.505 [160.7525; 494.4125]	0.12939	0.24
MIP-1α	32.72 [28.025; 46.8975]	152.61 [80.11; 229.3975]	0.00488	0.022
MIP-1β	75.515 [52.3025; 106.01]	339.915 [267.36; 417.425]	0.03418	0.091
PDGF-AA	16.875 [10.9775; 24.185]	21.31 [16.48; 45.2175]	0.01221	0.041
PDGF-AB/BB	32.915 [12.2775; 42.6975]	26.48 [14.15; 41.905]	0.67725	0.8
RANTES	16.44 [10.9; 22.48]	19.535 [15.88; 46.565]	0.05225	0.12
sCD40L	48.035 [16.8475; 83.5625]	43.31 [18.385; 56.31]	0.73340	0.84
TGF-α	6.505 [3.5925; 9.285]	3.075 [2.2675; 5.665]	0.05225	0.12

TNF-α	11.815 [6.885; 18.4575]	26.725 [16.7275; 63.095]	0.01611	0.05
TNF-β	4.555 [1.11875; 7.635]	4.175 [1.59875; 5.925]	0.62483	0.78
VEGF	51.77 [28.85; 68.725]	193.155 [49.48; 582.5075]	0.01128	0.041

Green - *p*, *p*. adj. < 0.05. Culture medium was replaced 1 h after infection and then collected on days 4, 7 and 10. Measurement of cytokines was performed for each time point separately, then the results for days 4, 7 and 10 were included in one sample and analyzed with the Wilcoxon signed-rank test. Final sample set included 24 values (12 control, 12 experiment values) for each cytokine.

Supplementary Table S6. Dynamics of cytokine concentrations in lung tissue explants infected with SARS-CoV-2.

Cytokine	Day	Control	SARS-CoV-2
EGF	4	9.33 [3.875; 14.7525]	12.58 [9.1; 14.8]
EGF	7	14.83 [6.89; 24.2175]	10.315 [7.32; 22.4675]
EGF	10	10.49 [8.1975; 12.345]	9.805 [6.8275; 12.0975]
Eotaxin	4	14.01 [9.9925; 18.705]	13.465 [11.3625; 18.8425]
Eotaxin	7	17.44 [12.9875; 21.325]	13.17 [11.1725; 23.6375]
Eotaxin	10	11.61 [10.1925; 55.5625]	13.51 [11.4275; 15.645]
FGF-2	4	36.395 [14.15; 61.0475]	55.78 [40.57375; 63.1675]
FGF-2	7	56.41 [29.60125; 92.3575]	44.695 [33.36625; 85.8975]
FGF-2	10	41.33 [35.6675; 47.6325]	39.035 [25.065; 51.1975]
Flt-3L	4	18.275 [7.73; 29.6375]	21.7 [13.3175; 32.8275]
Flt-3L	7	26.31 [15.1375; 42.36]	16.595 [13.3375; 39.3225]
Flt-3L	10	21.395 [17.4625; 23.6325]	17.035 [12.995; 19.34]
Fractalkine	4	214.96 [109.525; 314.1525]	165.16 [121.01; 275.02]
Fractalkine	7	259.18 [155.365; 381.0875]	144.345 [109.785; 415.8175]
Fractalkine	10	108.005 [99.1525; 159.815]	190.445 [86.99; 277.7075]
G-CSF	4	12176.855 [6664.675; 21030.0525]	37385.975 [25454.87; 58715.7075]
G-CSF	7	9595.81 [7038.21; 13946.855]	69269.41 [41902.6675; 114280.76]

G-CSF	10	10033.635 [8702.3625; 23646.845]	61350.27 [43213.2; 107929.7925]
GM-CSF	4	24.5 [16.305; 42.8425]	177.21 [60.71; 380.8675]
GM-CSF	7	63.195 [23.53; 108.12]	294.795 [205.0125; 592.9325]
GM-CSF	10	158.225 [93.52; 258.465]	327.34 [296.52; 860.7975]
GRO-α	4	4341.69 [2589.415; 7398.26]	13332.51 [8528.84; 18410.0275]
GRO-α	7	4345.275 [2937.06; 6367.55]	27810.695 [17195.11; 38715.755]
GRO-α	10	7178.535 [4406.3125; 15293.8375]	23839.225 [15587.375; 40700.1125]
IFN-α2	4	28.79 [13.3075; 45.55]	37.76 [27.02625; 47.0125]
IFN-α2	7	39.85 [21.995; 63.3525]	29.085 [19.6025; 72.445]
IFN-α2	10	31.58 [25.6725; 36.4225]	28.495 [22.545; 30.575]
IFN-γ	4	8.39 [2.985; 13.51]	13.6 [7.4475; 21.2]
IFN-γ	7	12.905 [5.38; 26.6575]	54.98 [19.1775; 108.4425]
IFN-γ	10	9.405 [8.3525; 9.725]	116.365 [18.0425; 236.045]
IL-10	4	11.235 [4.9475; 17.735]	50.735 [40.7275; 73.2]
IL-10	7	13.705 [5.6225; 27.8225]	28.82 [24.8875; 58.26]
IL-10	10	15.13 [8.7075; 24.1675]	18.29 [13.7925; 24.1675]
IL-12p40	4	21.46 [10.115; 34.575]	24.47 [16.73; 34.88]
IL-12p40	7	33.28 [18.3725; 59.55]	22.36 [17.3925; 72.48]
IL-12p40	10	20.335 [17.93; 22.13]	22.92 [18.1225; 24.995]
IL-12p70	4	10.97 [3.645; 18.37]	9.65 [5.7375; 18.61]

IL-12p70	7	14.2 [7.5575; 26.38]	7.475 [5.5425; 31.5275]
IL-12p70	10	7.565 [5.7675; 10.51]	8.31 [6.0275; 9.515]
IL-13	4	14.535 [7.2025; 22.035]	13.82 [8.805; 22.45]
IL-13	7	20.46 [11.3375; 31.045]	10.67 [8.325; 28.2625]
IL-13	10	9.065 [7.4425; 12.6875]	12.975 [8.3625; 16.2025]
IL-15	4	6.275 [2.165; 11.415]	8.065 [5.0075; 11.68]
IL-15	7	12.55 [6.8525; 20.615]	8.645 [6.235; 26.2325]
IL-15	10	10.95 [9.4775; 12.1225]	8.81 [7.215; 12.325]
IL-17A	4	4.43 [1.6925; 6.955]	5.325 [2.8325; 10.34]
IL-17A	7	6.185 [4.06; 10.31]	5.4 [3.045; 18.4275]
IL-17A	10	3.29 [2.35; 5.075]	5.6 [3.1425; 9.75]
IL-1α	4	3.845 [1.3075; 6.7325]	7.005 [3.7425; 10.06]
IL-1α	7	8.83 [3.7825; 15.6475]	8.73 [7.9475; 22.355]
IL-1α	10	10.05 [8.045; 12.49]	11.76 [10.6; 17.39]
IL-1β	4	16.75 [15.1925; 18.795]	23.13 [19.1925; 31.565]
IL-1β	7	17.865 [15.2975; 23.2]	19.71 [17.61; 29.75]
IL-1β	10	18.535 [16.5875; 21.295]	18.16 [17.0925; 20.565]
IL-1RA	4	23.485 [10.2275; 46.685]	38.1 [13.475; 60.1425]
IL-1RA	7	169.455 [95.8025; 253.14]	19.075 [14.9925; 80.9325]
IL-1RA	10	150.7 [46.9525; 306.6925]	17.72 [14.47; 29.565]

IL-2	4	1.825 [0.57625; 3.055]	2.065 [0.83; 3.2025]
IL-2	7	2.865 [0.7425; 6.06]	2.265 [1.9675; 7.6175]
IL-2	10	2.73 [1.9975; 3.14]	2.235 [1.9175; 2.8425]
IL-4	4	87.35 [22.4025; 154.1275]	139.26 [124.11; 157.0975]
IL-4	7	150.56 [37.0825; 297.3925]	158.14 [139.5; 350.3525]
IL-4	10	140.96 [105.035; 162.64]	153.04 [135.33; 177.115]
IL-5	4	1.1675 [0.335; 2.4925]	1.965 [1.18625; 3.14]
IL-5	7	2.2375 [0.31875; 5.325]	2.94 [0.935; 9.8675]
IL-5	10	1.86 [1.5175; 4.9575]	3.91 [1.68875; 7.655]
IL-6	4	23272.46 [15332.4075; 33028.6275]	39919.085 [29810.4675; 58070.3075]
IL-6	7	25337.68 [17476.68; 37426.65]	73532.5 [54099.5025; 218188.6575]
IL-6	10	30235.01 [20390.5875; 186095.385]	94120.09 [59547.115; 244651.14]
IL-7	4	69.005 [61.84; 74.77]	69.905 [69.385; 76.1925]
IL-7	7	71.135 [61.7125; 79.175]	69.29 [66.8275; 83.7825]
IL-7	10	67.255 [65.5875; 70.1925]	69.065 [66.0725; 73.1575]
IL-8	4	34037.19 [17038.07; 58515.315]	54559.905 [46126.3575; 63152.6225]
IL-8	7	27543.53 [16026.955; 44421.3425]	99067.825 [74635.565; 187365.875]
IL-8	10	21444.135 [13208.51; 47209.7]	104474.08 [64490.8825; 218706.685]
IL-9	4	3.895 [1.14; 6.465]	3.035 [1.5275; 5.7475]

IL-9	7	4.59 [1.9475; 8.395]	3.425 [2.21625; 12.185]
IL-9	10	2.49 [1.7025; 3.38]	4.065 [2.53875; 5.5275]
IP-10	4	47.075 [43.4275; 57.425]	108.295 [42.75; 298.5825]
IP-10	7	277.855 [169.9575; 432.2375]	2588.16 [899.18; 5119.6825]
IP-10	10	721.885 [392.0025; 1026.1675]	5325.54 [1082.2825; 9712.805]
MCP-1	4	25625.22 [16815.6775; 35790.73]	33380.085 [20807.135; 59013.875]
MCP-1	7	77577.05 [52878.215; 110805.0325]	103702.41 [64186.485; 192317.45]
MCP-1	10	106380.41 [80196.6525; 180893.3975]	126274.78 [61503.52; 229645.5575]
MCP-3	4	37.275 [8.5625; 84.3775]	146.63 [44.6725; 285.13]
MCP-3	7	324.985 [229.6225; 366.2]	870.42 [449.2875; 1339.9925]
MCP-3	10	499.27 [388.2725; 777.155]	1120.28 [497.07; 5114.4525]
MDC	4	70.09 [33.3925; 108.73]	89.95 [70.91; 121.7775]
MDC	7	291.93 [176.68; 382.3]	336.36 [298.26; 417.11]
MDC	10	554.27 [413.535; 748.4825]	605.68 [400.595; 842.1975]
MIP-1α	4	34.915 [29.855; 39.1375]	328.225 [242.9525; 447.16]
MIP-1α	7	30.555 [25.1175; 39.415]	152.61 [135.6475; 161.585]
MIP-1α	10	42.875 [26.27; 79.17]	62.945 [61.2125; 69.75]
MIP-1β	4	70.81 [49.5625; 85.98]	378.9 [267.36; 448.63]
MIP-1β	7	75.515 [59.8175; 104.7125]	383.825 [292.3625; 417.425]

MIP-1β	10	153.485 [55.39; 391.105]	322.42 [258.6925; 341.8875]
PDGF-AA	4	16.875 [12.785; 34.2]	30.99 [18.2325; 66.22]
PDGF-AA	7	25.53 [19.6025; 35.845]	38.12 [22.765; 60.78]
PDGF-AA	10	12.685 [9.5275; 15.1975]	15.315 [12.0625; 19.1025]
PDGF-AB/BB	4	25.64 [10.4375; 42.94]	30.705 [21.8175; 47.7875]
PDGF-AB/BB	7	35.64 [20.485; 52.92]	24.135 [19.055; 81.145]
PDGF-AB/BB	10	27.35 [14.2425; 40.225]	28.385 [13.5175; 41.905]
RANTES	4	18.615 [13.05; 23.0175]	17.505 [12.33625; 25.095]
RANTES	7	20.665 [12.8; 26.38]	23.9 [18.925; 37.6775]
RANTES	10	12.85 [10.9; 16.37]	37.28 [16.6575; 62.9975]
sCD40L	4	44.71 [8.6775; 83.5625]	54.765 [32.215; 82.14]
sCD40L	7	67.675 [27.8575; 137.555]	39.75 [26.875; 185.9075]
sCD40L	10	48.035 [36.1275; 54.65]	34.72 [16.4275; 49.0125]
TGF-α	4	3.23 [2.275; 4.3]	3.255 [2.595; 3.87]
TGF-α	7	9.725 [7.11; 11.485]	5.02 [2.6275; 9.66]
TGF-α	10	7.655 [5.895; 11.165]	2.73 [2.15; 4.68]
TNF-α	4	9.76 [4.4975; 15.3875]	65.34 [58.1225; 80.18]
TNF-α	7	12.645 [7.605; 18.1425]	22.815 [19.26; 37.1975]
TNF-α	10	15.33 [8.5225; 22.575]	11.445 [6.4375; 19.0075]

TNF-β	4	4.0525 [0.755; 7.635]	5.615 [2.94125; 8.055]
TNF-β	7	8.64 [2.50625; 17.6475]	4.38 [2.72375; 18.4125]
TNF-β	10	4.555 [2.905; 5.9575]	3.28 [1.59875; 4.715]
VEGF	4	41.825 [16.215; 68.725]	59.5 [33.46; 89.8925]
VEGF	7	52.43 [31.58125; 96.6975]	347.04 [208.225; 479.2775]
VEGF	10	48.34 [32.93; 215.615]	638.565 [438.69; 1083.355]
